# Selective vulnerability of cerebral vasculature to *NOTCH3* variants in small vessel disease and rescue by phosphodiesterase-5 inhibitor

**DOI:** 10.1126/sciadv.aeb1134

**Published:** 2026-04-03

**Authors:** Xiangjun Zhao, Chaowen Yu, Antony Adamson, Aite Zhao, Huiyu Zhou, Pankaj Sharma, Tao Wang

**Affiliations:** ^1^ Division of Evolution, Infection and Genomic Sciences, School of Biological Sciences, Faculty of Biology, Medicine, and Health, The University of Manchester, Manchester, UK.; ^2^ Children’s Hospital of Chongqing Medical University, Chongqing 400014, P.R. China.; ^3^ Genome Editing Unit, Faculty of Biology, Medicine and Health, The University of Manchester, Manchester, UK.; ^4^ College of Computer Science and Technology, Qingdao University, Qingdao, P.R. China.; ^5^ School of Computing and Mathematical Sciences, University of Leicester, Leicester, UK.; ^6^ Institute of Cardiovascular Research, Royal Holloway, University of London, Ashford and St Peter’s NHS Foundation Trust, and Imperial College Healthcare NHS Trust, London, UK.; ^7^ Manchester Centre for Genomic Medicine, Manchester University NHS Foundation Trust, Manchester, UK.; ^8^ Geoffrey Jefferson Brain Research Centre, Manchester Academic Health Science Centre, Northern Care Alliance and University of Manchester, Manchester, UK.

## Abstract

*NOTCH3* variants cause CADASIL (cerebral autosomal dominant arteriopathy and subcortical infarcts and leukoencephalopathy), the most common monogenetic form of small vessel disease (SVD) and vascular dementia (VaD). The molecular mechanisms driving CADASIL pathogenesis remain poorly understood, and no specific treatments are currently available. *NOTCH3* is mainly expressed in vascular smooth muscle cells (VSMCs) that arise from different embryonic origins. Using human induced pluripotent stem cell (iPSC) models, we generated origin-specific VSMCs and found that cerebral, but not peripheral, VSMC mimics are selectively vulnerable to *NOTCH3* variants. CADASIL iPSC–derived brain-specific VSMCs acquired a synthetic phenotype, accompanied with extensive extracellular matrix accumulation and impaired cell adhesion leading to anoikis. Furthermore, an endothelial-independent nitric oxide signaling was substantially impaired in CADASIL iPSC–derived VSMCs. Phosphodiesterase-5 inhibition successfully reversed the functional abnormality and survival of mutant VSMCs. Our findings uncovered mechanistic insights and suggest a viable therapeutic strategy for *NOTCH3*-associated SVD/VaD, reinforcing the value of patient-specific iPSCs for disease modeling and potential drug discovery.

## Teaser

iPSC models uncovered disease insights for *NOTCH3*-SVD, CADASIL, with successful treatment using PDE5 inhibitor sildenafil.

## INTRODUCTION

Small vessel disease (SVD) is the leading cause of vascular dementia (VaD) and the second commonest cause of dementia after Alzheimer’s disease (*1*). Genetic factors play an important role in the susceptibility, development, and progression of SVD (*2*). CADASIL (cerebral autosomal dominant arteriopathy and subcortical infarcts and leukoencephalopathy) is the most common form of genetic SVD (*3*, *4*). Patients with CADASIL typically experience recurrent strokes, and many with migraine and mood disturbance gradually progressing to cognitive impairment leading to VaD (*3*, *4*). As a systemic vasculopathy that affects small vessels throughout the body, its brain-specific clinical manifestations are intriguing. The exact molecular mechanisms by which *NOTCH3* variants lead to vascular pathology and neurological symptoms remain unclear; consequently, no specific or effective treatments are currently available.

The causative gene for CADASIL is *NOTCH3* that is predominantly expressed in arterial vascular smooth muscle cells (VSMCs) (*5*, *6*). NOTCH3 signaling promotes VSMC differentiation and proliferation, inhibits VSMC migration, and protects VSMCs from apoptosis, playing a key role in maintaining vascular homeostasis (*7*, *8*). The main pathology of CADASIL is VSMC degeneration in small arteries leading to fibrosis, luminal narrowing, and wall thickening of the vessel (*3*, *9*). NOTCH3 is a single-pass transmembrane receptor with a large extracellular domain (N3ECD) containing 34 epidermal growth factor (EGF)–like repeats, each with six cysteine residues forming three disulfide bonds. Nearly all CADASIL-associated *NOTCH3* variants result in the gain or loss of a cysteine residue, producing an odd number of cysteines within the affected EGF repeat (*10*), which likely affects normal N3ECD folding leading to its pathological accumulation. In addition, a unique feature of CADASIL seen under transmission electron microscopy (TEM) is the deposition of a granular osmophilic material (GOM) around VSMCs and pericytes in small arteries (*10*, *11*). While the main component of GOM was identified to be misfolded N3ECD with several extracellular matrix (ECM) proteins (*12*, *13*), the complete composition and structure of the GOM are not fully defined. Protein accumulation suggests a toxic gain of function; however, given that Notch signaling cross-talks with multiple signaling pathways (*14*, *15*), it remains unclear whether and how the mutant *NOTCH3* disrupts the cross interactions and contributes to the development of CADASIL pathologies. Elucidating these mechanisms will have important therapeutic implications.

VSMCs exhibit developmental heterogeneity, originating from both neural crest and mesodermal subdomains (*16*). VSMCs in cerebral arteries are mainly derived from neural crest cells, those in coronary arteries originate from lateral plate mesoderm via the epicardium, and VSMCs in peripheral arteries arise mainly from paraxial mesoderm. The origin-specific VSMCs have differential responses to growth factors like angiotensin II (*17*) and show varying disease susceptibilities, independent of hemodynamic factors (*18*, *19*). *NOTCH3* is broadly expressed throughout the vascular system, but the susceptibility of the origin-specific VSMCs to *NOTCH3* variants are unexplored, leaving a key knowledge gap in understanding the brain-predominant clinical features observed in CADASIL. Furthermore, VSMCs are not terminally differentiated and exhibit phenotypic plasticity in response to various stimuli (*20*). The transition of VSMCs from a contractile to a synthetic phenotype is a critical process in the development of common cardiovascular diseases like atherosclerosis and vascular remodeling. Synthetic VSMCs increase the production of ECM proteins, growth factors, and cytokines and are characterized by increased proliferation and migration (*16*). Despite evidence of impaired vasoreactivity in both patients with CADASIL and transgenic mouse models (*21*, *22*), the VSMC phenotype in CADASIL has not been systematically studied.

In the past decades, CADASIL mouse models have uncovered valuable insights into molecular mechanisms of the disease. However, most of these animal models failed to recapitulate the full range of neurological symptoms observed in human patients, although they successfully phenocopied key pathological features of CADASIL including VSMC degeneration, GOM deposition and Notch3 accumulation (*23*). Given that 90% of drugs that pass preclinical testing in animal models ultimately fail to reach patients (*24*), there is a critical need for human disease models as complimentary tools. In this regard, induced pluripotent stem cells (iPSCs) derived from patients with CADASIL provide a valuable platform for better understanding of this condition and accelerating therapeutic discovery.

We previously established iPSC models of CADASIL from patient skin biopsies, revealing that CADASIL VSMCs failed to stabilize angiogenic capillary structures and support blood-brain barrier integrity (*25*, *26*). In this study, we generated developmental origin-specific VSMCs from iPSCs and identified a selective vulnerability of neural crest–derived VSMCs to *NOTCH3* variants, characterized by their phenotype switching, altered ECM composition, and reduced cell survival. The local nitric oxide (NO) signaling in the CADASIL VSMCs was compromised, and treatment with either an NO donor or phosphodiesterase-5 (PDE5) inhibitor (sildenafil) significantly reversed VSMC dysfunction and cell death, suggesting a viable therapeutic approach for this condition.

## RESULTS

### IPSC-derived brain-specific VSMCs are selectively vulnerable to *NOTCH3* variants in CADASIL

**Fig. 1.**
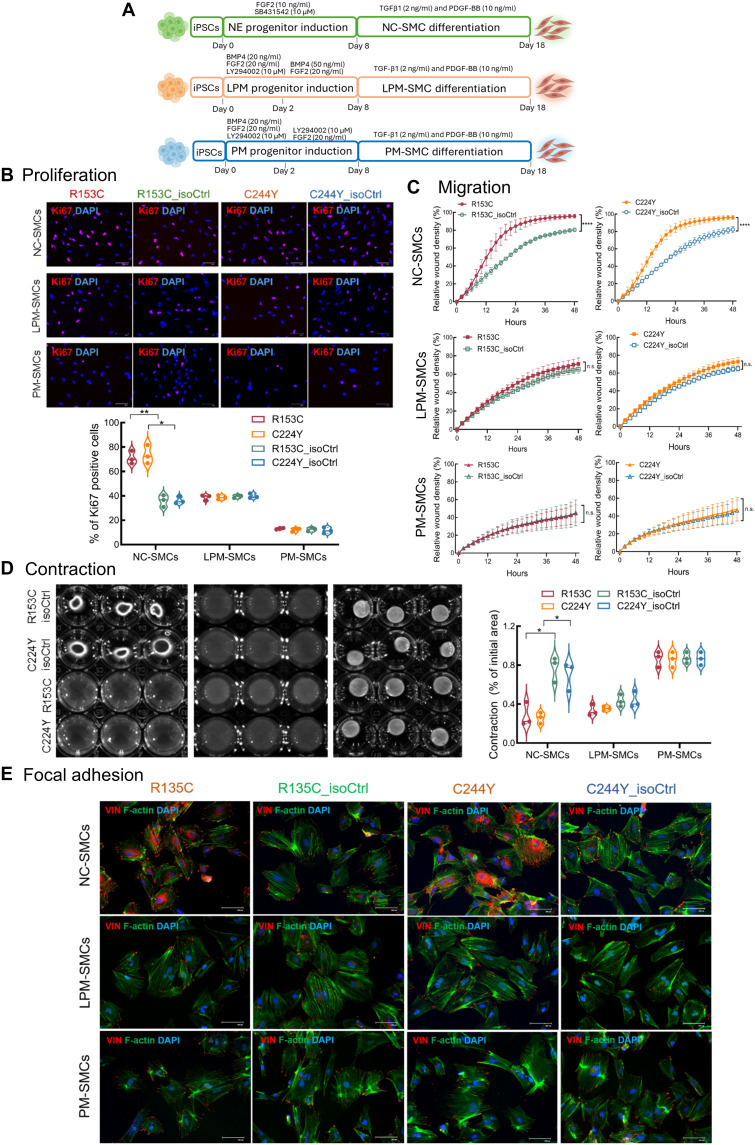
Functional comparison of lineage-specific VSMCs derived from iPSCs of patients with CADASIL and isoCtrls. (**A**) Schematic illustration of differentiation protocols used to generate VSMCs from iPSCs derived from two patients with CADASIL (R153C and C224Y) and their isoCtrls (isoCtrl) via neural crest (NC), lateral plate mesoderm (LPM), and paraxial mesoderm (PM) lineages, namely, NC-SMCs, LPM-SMCs, and PM-SMCs, respectively. (**B**) Cell proliferation was determined by IF staining for Ki67 (red). Nuclei were counterstained by 4′,6-diamidino-2-phenylindole (DAPI) (blue). Quantification shown below. Scale bars, 100 μm. (**C**) Cell migration assessed by wound healing assay using Incucyte live-cell imaging system. n.s., not significant. (**D**) Contractile function evaluated by collagen I gel contraction assay. Quantifications shown on the right. (**E**) Focal adhesions visualized by immunostaining for vinculin (red) and F-actin (green). Scale bars, 100 μm. Data are means ± SEM from three independent iPSC differentiations (*n* = 3). Unpaired Student’s *t* test was used for (B) and (D). Two-way analysis of variance (ANOVA) followed by post hoc testing was applied to (C). *P ≤ 0.05, **P ≤ 0.01, ****P ≤ 0.0001. Diagram (A) was created in BioRender. Wang, T. (2025) https://BioRender.com/m3dywe8.

To determine whether VSMCs in the brain vasculature have specific susceptibility to the *NOTCH3* variants in CADASIL that change cell behavior and functions, we differentiated iPSCs into VSMCs via neural crest, lateral plate mesoderm, and paraxial mesoderm, namely, NC-SMCs, LPM-SMCs, and PM-SMCs with each representing VSMCs of the brain, the heart, and peripheral, respectively, using established methods (*27*, *28*) with modifications (Fig. 1A).

We first used a wild-type iPSC line to validate features of the origin-specific iPSC-derived VSMCs. During the course of differentiation, pluripotency markers were down-regulated, and developmental lineage-specific markers *TFAPA2* (neural crest), *ISL1* (lateral plate mesoderm), and *TBX6* (paraxial mesoderm) were significantly up-regulated at the corresponding progenitor stages (fig. S1A). The three subtypes of iPSC-derived VSMCs all expressed VSMC-specific markers including *ACTA2*/α-smooth muscle actin (α-SMA), *CNN1*/calponin, *MYH11*/SMMHC, and *TAGLN*/SM22α as determined by reverse transcription quantitative polymerase chain reaction (RT-qPCR) (fig. S1A) and immunofluorescence (IF) staining (fig. S1B). Meanwhile, each type of the VSMCs expressed the corresponding lineage-specific markers: *SEMA3α* and *MEF2c* for NC-SMCs, *ISL1* and *HAND1* for LPM-SMCs, and *HOXA10* and *HOXC6* for PM-SMCs (fig. S1C) (*29*–*31*). Compared with PM-SMCs, NC-SMCs and LPM-SMCs exhibited significantly higher proliferation and migration rates, with the NC-SMCs showing the greatest levels, as measured by Ki67 IF staining (fig. S2A) and wound healing assay (fig. S2B), respectively. In contrast, PM-SMCs exhibited the highest contractility, and the LPM-SMCs were least contractile as measured by collagen I contraction assay (fig. S3A). These behavioral and functional properties of the origin-specific iPSC-VSMCs align well with findings reported on primary VSMCs obtained from various vascular locations (*32*, *33*). In addition, we found that the NC-SMCs exhibited the highest number of focal adhesions, followed by the LPM-SMCs, as measured by vinculin staining (fig. S3B), suggesting a more active interaction between VSMCs and the matrix in the vasculature of the central nervous system (CNS). PM-SMCs displayed the least number of focal adhesions (fig. S3B).

Having validated the identity of the lineage-specific iPSC-derived VSMCs, we compared the behavior and basic functionalities between VSMCs derived from iPSCs of two patients with CADASIL (R153C and C224Y) and their corresponding isogenic controls (isoCtrls) that we recently generated using CRISPR-Cas9 gene editing (fig. S4). Cell proliferation, measured by Ki67 staining, revealed a significant increase in NC-SMCs derived from the two CADASIL iPSC lines as compared with their isoCtrls (Fig. 1B). Neither LPM-SMCs nor PM-SMCs from the two CADASIL iPSC lines had significantly altered their proliferation rate, suggesting a specific change in VSMCs in the CADASIL brain. Migration assay using the IncyCyte wound healing system further demonstrated that the CADASIL NC-SMCs, but not the LPM-SMCs nor PM-SMCs, had significantly increased migration relative to isoCtrls (Fig. 1C and fig. S5), supporting a selective susceptibility of CNS VSMCs to the CADASIL *NOTCH3* variants.

In addition to the behavioral changes, CADASIL NC-SMCs exhibited significantly impaired contractile function in the collagen I gel contraction assay, whereas the LPS-SMCs and PM-SMCs showed no difference from their isoCtrls (Fig. 1D). Vinculin immunostaining revealed increased but disorganized focal adhesions in CADASIL NC-SMCs, along with diffused cytosolic vinculin signals (Fig. 1E and fig. S6), indicative of potential defects in vinculin activation and membrane recruitment (*34*), likely disrupting cytoskeletal organization (*35*).

Together, results from comparing the behavior and function of the developmental lineage-specific VSMCs derived from iPSCs strongly suggest a brain-specific vascular vulnerability to the CADASIL *NOTCH3* variants (Fig. 1). In the following experiments, the iPSC NC-SMC model, namely, iVSMCs, was exclusively used to study disease mechanisms.

### CADASIL iVSMCs undergo phenotype switching from contractile to synthetic state

**Fig. 2.**
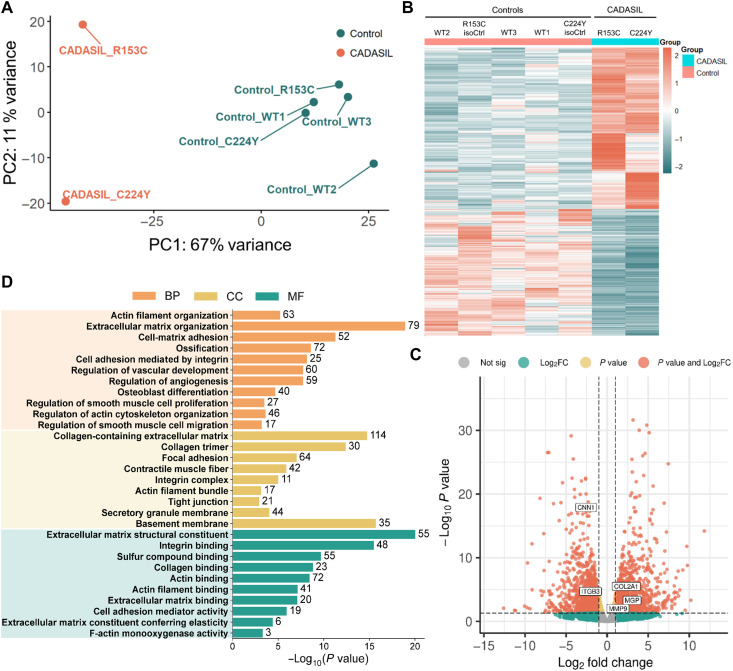
Transcriptomic analysis of iVSMCs from iPSCs of patients with CADASIL and isoCtrls. IPSCs from two patients with CADASIL (R135C and C224Y), their respective isoCtrls, and three wild-type iPSC control lines (WT1 to WT3) were differentiated into iVSMCs via neural crest lineage and subjected to bulk RNA-seq. (**A**) Principal component analysis (PCA) plot revealed clustering based on genotype. (**B**) Heatmap of differentially expressed genes (DEGs) between patient-derived iVSMCs and controls (2× isoCtrls and 3× WT controls). (**C**) Volcano plot displaying DEGs between patients with CADASIL (R135C and C224Y) and their isoCtrls (R153C_isoCtrl and C224Y_isoCtrl). (**D**) GO term enrichment analysis of DEGs. BP, biological processes; CC, cellular components; MF, molecular functions.

To gain a more comprehensive understanding of genes and signaling pathways that are involved in the pathological changes in CADASIL and identify previously unknown disease mechanisms and therapeutic targets, we conducted bulk RNA sequencing (RNA-seq) on iVSMCs differentiated from two CADASIL iPSC lines (R153C and C224Y), two isoCtrls (R153C_isoCtrl and C224Y_isoCtrl), and three wild-type control lines (02C9, 02C3, and EIPL1). Principal components analysis (PCA) showed that PC1 explained 67% of the variability that mainly distinguished the CADASIL samples from all the controls (Fig. 2A). Differential expression analysis revealed 4161 differentially expressed genes (*P* value <0.05 and Log_2_^(Fold change)^ > 1), including 1932 up-regulated and 2229 down-regulated genes, between the CADASIL and control samples (Fig. 2B). As the isoCtrls and wild-type controls clustered closely on the PCA plot (Fig. 2A), thus, only the isoCtrls were used in the subsequent data analysis to more accurately identify the impact by the *NOTCH3* variants while minimizing interindividual variability (Fig. 2C). Gene ontology (GO) analysis of biological process, cellular components, and molecular function terms highlighted alterations related to ECM organization, focal adhesion, smooth muscle cell proliferation, smooth cell migration, cell-matrix adhesion, actin filament organization, collagen-containing ECM, and regulation of vasculature development (Fig. 2D), suggesting a matrix-related pathology and phenotype changes of CADASIL VSMCs.

**Fig. 3.**
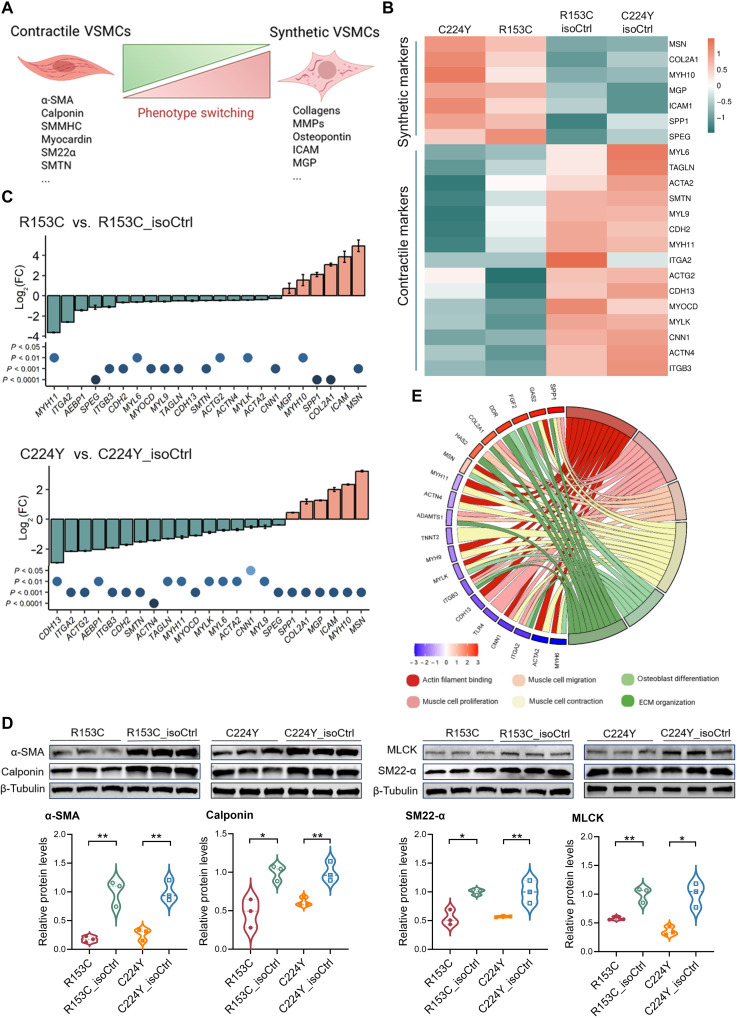
Phenotype change of iVSMCs derived from iPSCs of patients with CADASIL and isoCtrls. IPSCs from two patients with CADASIL (R135C and C224Y) and their isoCtrls (R153C_isoCtrl and C224Y_isoCtrl) were differentiated into iVSMCs via neural crest lineage. (**A**) Schematic illustration of VSMC phenotypic switching from a contractile to a synthetic state, highlighting changes of key marker gene expression. (**B**) Heatmap of differentially expressed VSMC contractile and synthetic marker genes identified by RNA-seq of iVSMCs from two patients with CADASIL and their isoCtrls. (**C**) RT-qPCR confirmation of altered expression of representative contractile and synthetic VSMC markers in CADASIL iVSMCs. Data are means ± SEM from three independent iPSC differentiations (*n* = 3), each with three technical replications. Fold changes were calculated individually for each gene and compared using unpaired Student’s *t* test. (**D**) Western blotting (WB) confirming down-regulation of contractile VSMC proteins (α-SMA, calponin, SM22α, and MLCK) in CADASIL iVSMCs versus their isoCtrls. Quantifications shown below the WB images. Data are means ± SEM from three independent iPSC differentiations (*n* = 3). Unpaired Student’s *t* test, *P ≤ 0.05, **P ≤ 0.01. (**E**) Chord diagram displaying associations between selected DEGs and VSMC phenotype-related GO. Diagram (A) was created in BioRender. Wang, T. (2025) https://BioRender.com/un8j2bx.

We next specifically analyzed the VSMC phenotype (Fig. 3A) on the iPSC CADASIL model. Heatmap from RNA-seq data shows down-regulation of a range of contractile marker genes including *MYH11*, *ACTA2*, *CNN1*, *TAGLN*, and *MYOCD* and up-regulation of many synthetic marker genes including *COL1A2*, *MMP9*, *SPP1*, *MYH10*, and *MSN* in the CADASIL iVSMCs (Fig. 3B) comparing with isoCtrls, which was confirmed by RT-qPCR (Fig. 3C). Western blotting further confirmed the decreased protein levels of key contractile markers α-SMA, myosin light chain kinase (MLCK), calponin, and SM22α in CADASIL iVSMCs (Fig. 3D). A chord diagram shows down-regulation of genes associated with GO terms of actin filament binding and muscle cell contraction (Fig. 3E). Increased proliferation and migration and reduced contractility are functional features of synthetic VSMCs (*36*), which we have demonstrated in CADASIL iVSMCs (EC-SMCs) above in Fig. 1. An additional migration assay using three-dimensional (3D) VSMC spheroids confirmed the enhanced migratory capacity of CADASIL iVSMCs (fig. S7). Together, results from gene expressions, protein levels, and functional assays suggest that CADASIL iVSMCs undergo phenotype switching from a contractile to a synthetic state.

### ECM composition and adhesion-actin coupling are severely disrupted in CADASIL iVSMCs, leading to cell death

**Fig. 4.**
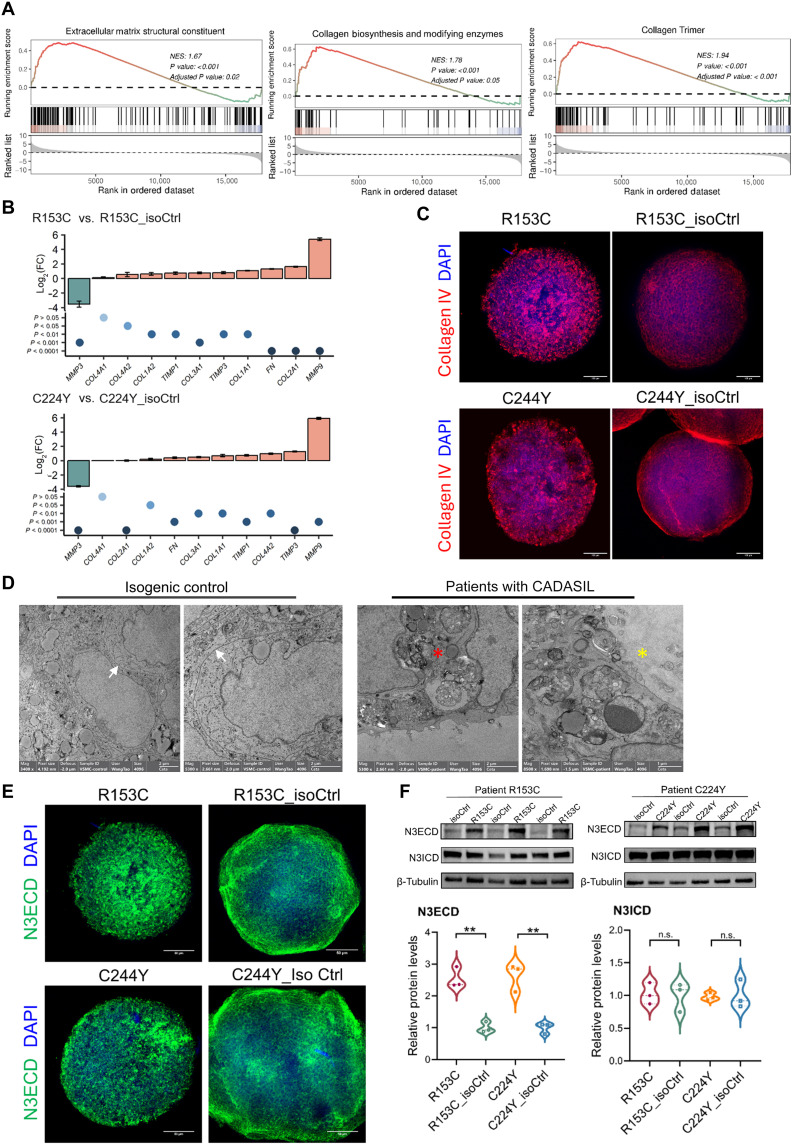
ECM accumulation in iVSMCs derived from iPSCs of patients with CADASIL and isoCtrls. IPSCs from two patients with CADASIL (R135C and C224Y) and their isoCtrls (R153C_isoCtrl and C224Y_isoCtrl) were differentiated into iVSMCs via neural crest lineage. (**A**) GSEA plot of RNA-seq data showing enrichment of GO terms relating to ECM structural constituents, collagen biosynthesis and modifying enzymes, and collagen trimer in CADASIL iVSMCs versus their isoCtrls. (**B**) RT-qPCR validation of differentially expressed major ECM-related genes in CADASIL iVSMCs versus their isoCtrls. Data are means ± SEM from three independent iPSC differentiations (*n* = 3). Fold changes for each gene were calculated separately and compared using unpaired Student’s *t* test. (**C**) IF staining of collagen IV in iVSMCs spheroids derived from CADASIL and isoCtrl iPSCs. Nuclei were counterstained with DAPI. Scale bar, 100 μm. (**D**) TEM images of iVSMCs showing ultrastructural differences between CADASIL and isoCtrl cells. White arrows, smooth cell membranes between adjacent cells. Red asterisk star, vanish of cell junctions and accumulation of extracellular materials in CADASIL iVSMCs. Yellow asterisk star, ECM accumulation. (**E**) IF staining of NOTCH3 extracellular domain (N3ECD) in iVSMCs spheroids derived from CADASIL and isoCtrl iPSCs. Nuclei were counterstained with DAPI. Scale bar, 100 μm. (**F**) WB of iVSMCs using antibodies against N3ECD or NOTCH3 intracellular domain (N3ICD). Quantifications shown below. Data are means ± SEM from three independent iPSC differentiations (*n* = 3). Unpaired Student’s *t* test, **P ≤ 0.01.

Under the GO term “extracellular matrix organisation (p = 3.49E-24),” there were around 150 differentially expressed between the CADASIL iVSMC and isoCtrls as shown on the heatmap (fig. S8A). Of the 150 genes, 43 genes were identified to be collagen related, with the majority of which were up-regulated (fig. S8B). We then conducted gene set enrichment analysis (GSEA) to uncover the coordinated change of genes involved in ECM function. GSEA revealed that gene sets relating to “ECM structural constituent,” “collagen biosynthesis and modifying enzymes,” and “collagen trimer” were positively enriched in the CADASIL iVSMCs (Fig. 4A), suggesting matrix accumulation and its abnormal turnover in CADASIL. RT-qPCR results confirmed the significant up-regulation of key basement membrane components including collagen subtypes (*COL4A2*, *COL1A1*, *COL1A2*, *COL2A1*, and *COL3A1*), matrix metalloproteinase (MMP) (*MMP9*), tissue inhibitor of matrix metalloproteinases (TIMPs) (*TIMP1* and *TIMP3*), and *FN* (fibronectin) in the CADASIL iVSMCs compared with isoCtrls (Fig. 4B). MMP3 was consistently found down-regulated in the CADASIL iVSMCs (Fig. 4B). Considering the unique role of MMP3 that has much broader ECM substrates, including fibronectin and proteoglycans apart from collagens, than other MMPs and participates in proMMP activations (*37*), the reduced MMP3 may play a unique role in the ECM remodeling in CADASIL. Consistent with the transcriptomic and RT-qPCR results, spheroids formed from CADASIL iVSMCs displayed collagen IV protein accumulation (Fig. 4C). TEM images revealed large amount of matrix deposition (Fig. 4D), which is consistent with findings on autopsy samples of human patient (*38*). Accompanied with the matrix deposition, accumulation of NOTCH3 extracellular domain proteins, one of the hallmarks of CADASIL pathology, was also faithfully recapitulated on the iVSMC spheroids (Fig. 4E) and Western blotting (Fig. 4F). Despite accumulation, the mutant Notch3 had impaired CBF1/RBP-Jκ–mediated canonical Notch activity as determined by luciferase assay (fig. S9).

**Fig. 5.**
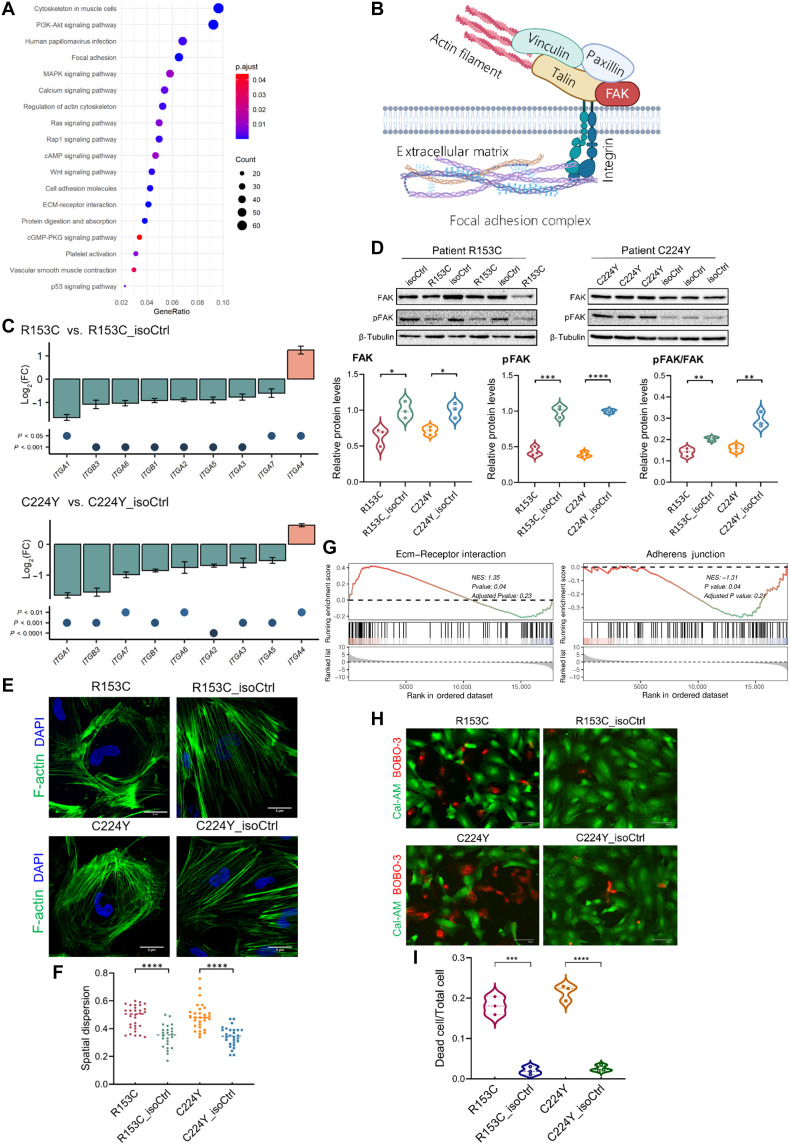
Disruption of cell-matrix and cell-cell interaction of CADASIL iVSMCs leading to cell death. IPSCs from two patients with CADASIL (R135C and C224Y) and their isoCtrls (R153C_isoCtrl and C224Y_isoCtrl) were differentiated into iVSMCs via neural crest lineage. (**A**) KEGG pathway enrichment analysis of DEGs from RNA-seq between iVSMCs from patients with CADASIL and isoCtrls. (**B**) Schematic illustration of key components of focal adhesion complex. (**C**) RT-qPCR analysis of the expression of integrin subtypes in iVSMCs derived from CADASIL and isoCtrl iPSCs. Data are presented as means ± SEM (*n* = 3). Fold changes were calculated for each gene and compared using unpaired Student’s *t* test. (**D**) WB analysis of total FAK and pFAK in iVSMCs derived from CADASIL and isoCtrl iPSCs. Quantifications shown at the bottom. Data are means ± SEM (*n* = 3). Unpaired Student’s *t* test, *P ≤ 0.05, **P ≤ 0.01, ***P ≤ 0.001, ****P ≤ 0.0001. (**E**) IF staining of F-actin in iVSMCs derived from CADASIL and isoCtrl iPSCs. Nuclei were counterstained by DAPI. Scale bar, 5 μm. (**F**) Artificial intelligence (AI)–assisted quantification of actin filament organization from (E) presented as spatial dispersion of actin structures. Unpaired Student’s *t* test, ****P ≤ 0.0001. (**G**) GSEA of RNA-seq data showing altered GO terms related to ECM-receptor interaction and adherens junctions in CADASIL iVSMCs versus isoCtrls. (**H**) Live/dead staining of iVSMCs using calcein-AM (Cal-AM; live; green) and BOBO-3 iodide (dead; red). Scale bar, 100 μm. (**I**) Quantification of the live/dead staining from (H). Data are means ± SEM from three independent differentiation (*n* = 3). Unpaired Student’s *t* test, ***P ≤ 0.001, ****P ≤ 0.0001. Results in (C), (D), (E), and (H) were all from at least three independent iPSC differentiations. Diagram (B) was created in BioRender. Wang, T. (2025) https://BioRender.com/tdigq98.

Kyoto Encyclopedia of Genes and Genomes (KEGG) analysis highlighted pathways relating to “cytoskeleton in muscle cells,” “focal adhesion,” “regulation of cytoskeleton,” “cell adhesion molecules,” and “ECM-receptor interaction” (Fig. 5A), suggesting changes in focal adhesion–actin coupling in CADASIL iVSMCs. Key components of focal adhesion include integrins, tailin, vinculin, paxillin, and focal adhesion kinase (FAK), which link actin cytoskeleton to ECM via integrins (Fig. 5B), playing a crucial role in cell-matrix interaction regulating cell behaviors and survival. We then quantified the expression of a range of integrin subunits (*ITGA1*, *ITGA2*, *ITGA3*, *ITGA4*, *ITGA5*, *ITGA6*, *ITGA7*, *ITGB1*, and *ITGB3*) in the iVSMCs using RT-qPCR and found all of which were significantly down-regulated in the CADASIL iVSMCs, except for *ITGA4* that was up-regulated in the mutant cells (Fig. 5C). *IGTA4* has a specific role in leukocyte adhesion and ECM remodeling in a form of α4β1 (*39*), and its up-regulation (Fig. 5C) warrants further investigation. Western blotting demonstrated significant reduction in total FAK and phosphorylated FAK (pFAK) as well as pFAK/FAK ratio in the CADASIL iVSMCs (Fig. 5D), suggesting a likely disruption of cell-matrix interaction that compromises cell survival (*40*). Fluorescent staining of F-actin cytoskeleton revealed disorganized actin fiber (Fig. 5E). To quantify this abnormality, we developed a computer vision algorithm based on Canny edge detection and measures of the disorder of samples, which demonstrated a significantly altered spatial dispersion of actin filaments in the CADASIL iVSMCs as compared with the isoCtrls (Fig. 5F), implicating compromised contractility, which is in line with the damaged contractile function (Fig. 1D) and reduced contractile markers (Fig. 3) in CADASIL iVSMCs as described earlier. In addition, GSEA showed significantly negative enrichment of “adherence junction” gene sets (Fig. 5G), suggesting additional defect in cell-cell interactions. This is also revealed on the TEM images, where smooth lines of cell membranes were observed between adjacent isoCtrl iVSMCs, which was hardly seen in the CADASIL iVSMCs (Fig. 4D). Instead, large amounts of cell debris and matrix were frequently observed between adjacent CADASIL iVSMCs (Fig. 4D). Together, our evidence on the disruption of interactions between both cell to matrix and cell to cell suggests a loss of cell anchorage, likely leading to anoikis (*41*). Live/dead assay demonstrated a significant increase of cell death in the CADASIL iVSMCs (Fig. 5, H and I).

### NO signaling is impaired in CADASIL iVSMCs

**Fig. 6.**
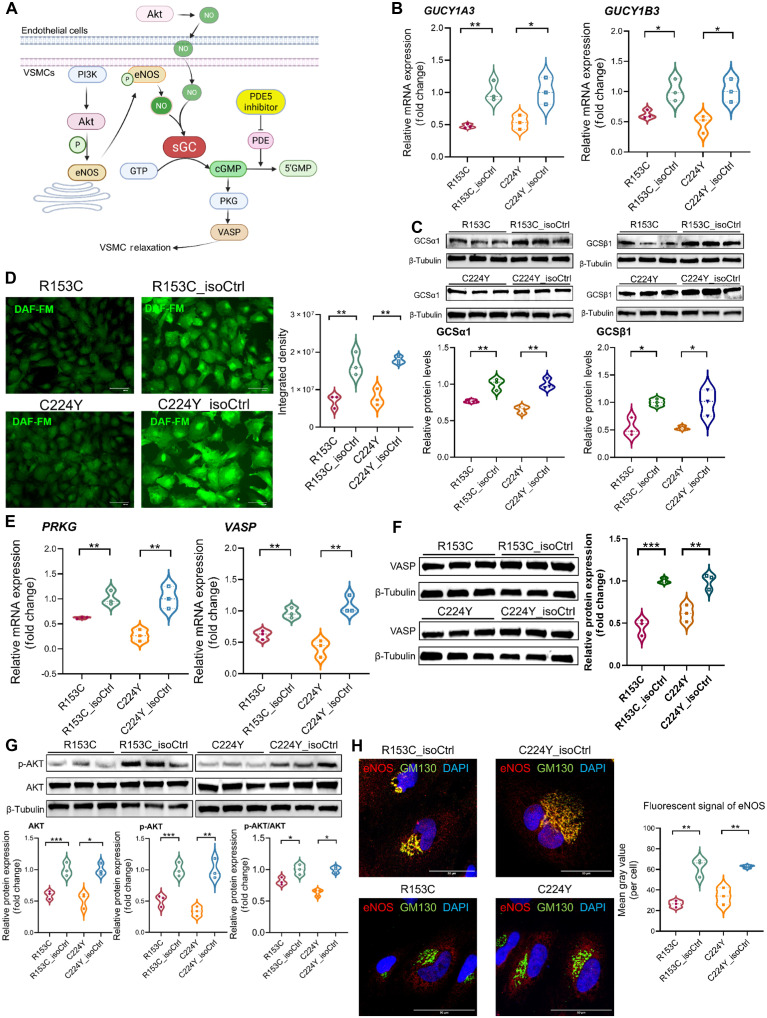
Impaired NO signaling in CADASIL iVSMCs. IPSCs from two patients with CADASIL (R135C and C224Y) and their isoCtrls (R153C_isoCtrl and C224Y_isoCtrl) were differentiated into iVSMCs via neural crest lineage. (**A**) Schematic illustration of the NO-sGC-cGMP-PKG signaling pathway. (**B**) RT-qPCR analysis of *GUCY1A3* and *GUCY1B3* expressions in iVSMCs derived from CADASIL and isoCtrl iPSCs. (**C**) WB analysis of GCSα1 and GCSβ1 protein levels in iVSMCs differentiated from CADASIL and isoCtrl iPSCs. (**D**) Cellular NO levels in iVSMCs determined by DAF-FM staining and their quantifications shown on the right. Scale bar, 100 μm. (**E**) RT-qPCR analysis of the expression of *PRKG* and *VASP* in iVSMCs derived from patients with CADASIL and their isoCtrl iPSCs. (**F** and **G**) WB determination of protein levels of VASP (F) and AKT and phosphorylated AKT (p-AKT) (G) in iVSMCs from CADASIL and isoCtrl iPSCs. (**H**) Immunofluorescent staining of eNOS (red) and costaining with Golgi marker GM130 (green) in iVSMCs. The fluorescence intensity of the eNOS signal was quantified and normalized to cell numbers (right). Scale bar, 50 μm. All quantitative data are presented as means ± SEM from three independent iPSC differentiations (*n* = 3). Statistical significance determined by unpaired Student’s *t* test, *P ≤ 0.05, **P ≤ 0.01, and ***P ≤ 0.001. Diagram (A) was created in BioRender. Wang, T. (2025) https://BioRender.com/fq74znk.

KEGG analysis of the RNA-seq data highlighted the “cGMP-PKG signaling pathway” (Fig. 5A). Given the fact that we had already observed reduced *GUCY1A1* [guanylate cyclase 1 subunit α1 (GCSα1)] and *GUCY1B1* [guanylate cyclase 1 subunit β1 (sGCβ1)] in CADASIL iVSMCs (Fig. 6, B and C) before the RNA-seq screening and the prior knowledge of the profound effects of NO in the regulation of VSMC contractility, proliferation, migration, ECM synthesis, and phenotype switching (*42*–*44*), we further explored the involvement of the NO–soluble guanylate cyclase (sGC)–guanosine 3′,5′-monophosphate (cGMP)–cGMP-dependent protein kinase (PKG) signaling (Fig. 6A) in VSMC pathology of CADASIL. We detected considerable levels of NO in the control iVSMCs with the absence of endothelial cells (ECs) as measured by 4-amino-5-methylamino-2′,7′-difluorofluorescein (DAF-FM) staining, which were significantly reduced in the CADASIL iVSMCs (Fig. 6D). GCSα1 and GCSβ1 are the two subunits of sGC that is the primary receptor of NO, which catalyze the synthesis of second messenger cGMP that activates PKG inducing VSMC dilation largely via vasodilator-stimulated phosphoprotein (VASP) (Fig. 6A) (*45*). The expression of *PRKG1* and *VASP* and the protein level of VASP were significantly down-regulated in CADASIL iVSMCs (Fig. 6, E and F). To understand the mechanism underlying the reduced NO in CADASIL iVSMCs, we found that endothelial NO synthase (eNOS) and AKT, a crucial activator of eNOS, were significantly reduced in CADASIL iVSMCs (Fig. 6G), while the inducible NOS (iNOS) remained unchanged (fig. S10). eNOS is primarily localized in the plasma membrane and Golgi apparitors, which plays an important role in regulating its enzymatic activity and the bioavailability of NO (*46*). We performed IF staining and found a notable difference in the subcellular localization patterns of eNOS between CADASIL and isoCtrl iVSMCs. In isoCtrl cells, a significant proportion of eNOS signals colocalized with the Golgi. In contrast, eNOS in CADASIL mutant cells showed minimal colocalization with Golgi and instead displayed weak and defused cytosolic signals (Fig. 6H). As the Golgi localization of eNOS is essential for its posttranslational modifications and activation, the finding likely underlies a compromised NO production (Fig. 6D) in CADASIL iVSMCs. Together, our results suggest the importance of the local NO-sGC-cGMP signaling in VSMCs independent of ECs, which was dysregulated in CADASIL iVSMCs.

### Treatment of iPSC CADASIL models by PDE5 inhibitor and NO donor significantly restored VSMC function

**Fig. 7.**
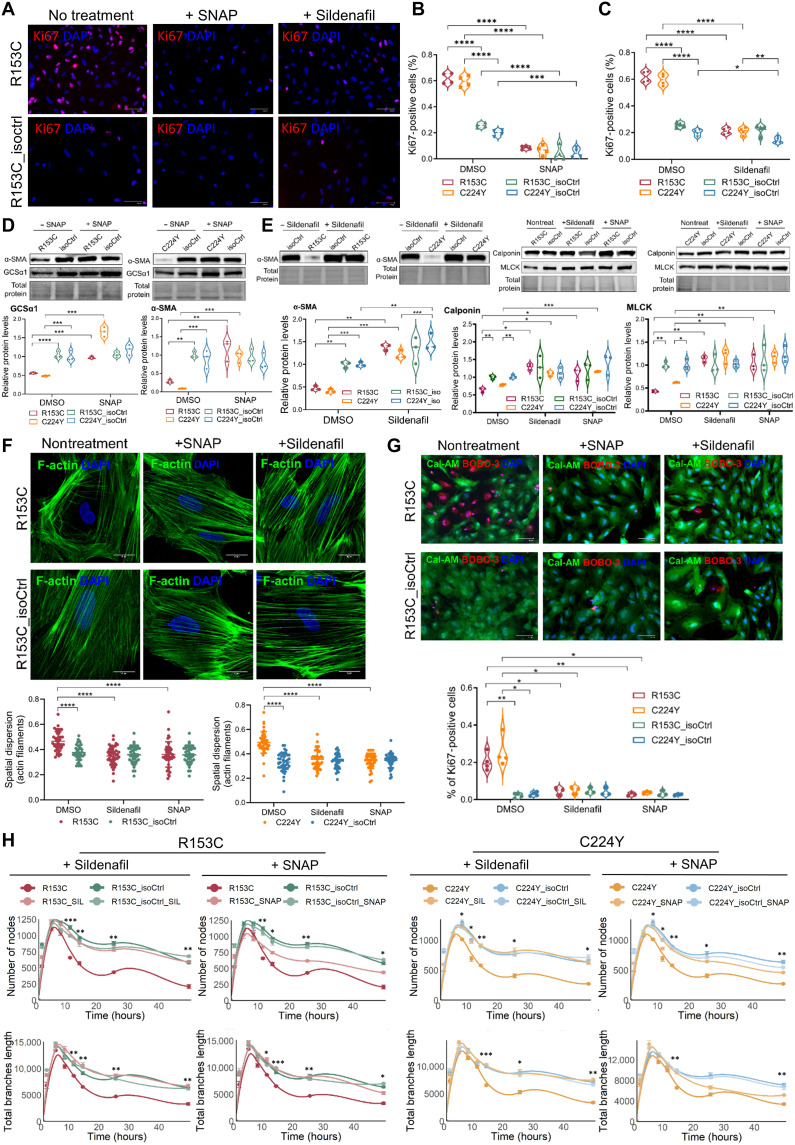
SNAP and sildenafil rescue abnormal phenotype and functions of CADASIL iVSMCs. IPSCs from two patients with CADASIL (R135C and C224Y) and their isoCtrls (R153C_isoCtrl and C224Y_isoCtrl) were differentiated into iVSMCs via neural crest lineage. (**A**) Immunofluorescent staining of Ki67 in iVSMCs with or without SNAP or sildenafil treatment. Nuclei were counterstained with DAPI. Scale bars, 100 μm. (**B** and **C**) Quantification of Ki67^+^ proliferative cells from (A). DMSO, dimethyl sulfoxide. (**D**) WB analysis of GCSα1 and α-SMA with or without SNAP treatment. (**E**) WB of α-SMA levels with or without sildenafil treatment and calponin and MLCK levels following SNAP or sildenafil treatment, respectively. Protein levels were normalized to total protein loading controls (representative loading areas shown below each blot). Qualifications of the WB results, normalized to total proteins, are shown below each WB image. (**F**) F-actin staining of iVSMCs treated with or without SNAP or sildenafil. Nuclei were counterstained by DAPI. Actin filament organization was analyzed using an AI-assisted algorithm and quantified as spatial dispersion (bottom). Scale bars, 5 μm. (**G**) Live/dead staining using Cal-AM (live; green) and BOBO-3 iodide (BOBO-3; dead; red) in iVSMCs. Quantification shown below. Scale bars, 100 μm. (**H**) In vitro angiogenesis assay using coculture of human umbilical cord ECs and iVSMCs in Matrigel with or without SNAP or sildenafil treatment. Quantification of “number of nodes” and “total branches length” was shown as the fitted smooth trend lines (LOESS/liner) with 95% confidence interval. All quantitative data are presented as means ± SEM from three independent iPSC differentiations (*n* = 3). Statistical analysis by two-way ANOVA followed by Tukey’s post hoc test, *P ≤ 0.05, **P ≤ 0.01, ***P ≤ 0.001, and ****P ≤ 0.0001 [the asterisk stars (*s) in (H) denoted significant differences of the treated versus nontreated samples of CADASIL iVSMCs at indicated time points].

Building upon our findings, we explored therapeutic potential by boosting the NO-sGC-cGMP signaling pathway to rescue the key pathological phenotypes of CADASIL iVSMCs. As proof-of-concept experiments, we first supplemented *S*-nitroso-*N*-acetylpenicillamine (SNAP), a synthetic NO donor that releases NO under physiological conditions, in cell culture and found that it significantly reversed the abnormal proliferation of CADASIL iVSMCs (Fig. 7, A and B, and fig. S11A) and moralized protein levels of GCSα1 and α-SMA (Fig. 7D). However, NO donors are usually unstable and their half-life varies. To achieve prolonged activation of NO signaling to benefit patients with CADASIL and also on the basis of the significant but mild reduction of cGC (*GUCY1A1* and *GUCY1B1*, Fig. 6C) in CADASIL iVSMCs, we applied the US Food and Drug Administration (FDA)–approved PDE5 inhibitor, sildenafil, that prevents the breakdown of sGC-generated cGMP and activates endogenous sGC-cGMP signaling (*47*). Results showed that sildenafil successfully reversed the hyperproliferation of CADASIL iVSMCs to the level of the isoCtrls (Fig. 7, A and C, and fig. S11A). The treatments also significantly increased the expression of contractile markers α-SMA, calponin, and MLCK in the CADASIL iVSMCs (Fig. 7, D and E). The disorganized actin cytoskeleton in CADASIL iVSMCs were significantly normalized by both SNAP and sildenafil treatments (Fig. 7F and fig. S11B). Both SNAP and sildenafil successfully prevented the cell death of the CADASIL iVSMCs (Fig. 7G and fig. S11C).

Linking to our previous finding that the CADASIL iVSMCs failed to stabilize the capillary tubule structure (*25*), we applied SNAP and sildenafil, respectively, during in vitro angiogenesis assay. Results showed that both SNAP and sildenafil could significantly prolong the EC tubule stability (Fig. 7H and figs. S12 and S13). Sildenafil outperformed SNAP as it could always stabilize the tubule structure to the isoCtrl level, suggesting the benefit of elevating endogenous cGMP over simply providing exogenous NO. Together, our results suggest a promising treatment of patients with CADASIL by PDE5 inhibitors to rescue VSMC function and prevent their degeneration seen in CADASIL small vessels.

## DISCUSSION

Using an iPSC model of CADASIL, we found that human iPSCs generated VSMCs via a developmental pathway mimicking the source of cerebral vasculature exhibit a unique susceptibility to CADASIL-associated *NOTCH3* mutations, compared with iPSC-derived VSMCs representing peripheral vascular sources. CADASIL iVSMCs displayed hyperproliferation, hypermigration, reduced contractility, and abnormal cell-matrix interactions. This study also provided a comprehensive and systemic evaluation of VSMC phenotype in CADASIL, revealing a phenotypic switch from a contractile to a synthetic state and reinforcing the role of ECM disorganization in CADASIL pathology. In addition, we provided evidence of disrupted cell-matrix and cell-cell adhesion of CADASIL iVSMCs, likely leading to a loss of cell anchorage and subsequent anoikis. Last, alterations in iVSMC-specific NO-sGC-cGMP signaling and phenotypic rescue of CADASIL iVSMCs by a PDE5 inhibitor, sildenafil, highlight a promising therapeutic avenue for CADASIL.

It is uncommon for CADASIL to primarily present as myocardial infarction or symptoms of peripheral vessel disease, albeit a systemic vasculopathy. The selective vulnerability of the CADASIL NC-SMCs offers one of the potential explanations of this paradox. Developmental origin-specific VSMCs exhibit distinct functionalities, including differences in growth behaviors and responses to stimuli (*17*, *32*, *33*, *48*, *49*). These differences are intrinsic and likely driven by unique gene expression profiles including the differential expressed *HOX* genes in lineage-specific VSMCs (*31*, *50*, *51*). For example, in a mouse model, when vessels prone to atherosclerosis were grafted to a low-pressure area of the vascular tree, they developed atherosclerosis in the absence of mechanical and hemodynamic factors (*52*), highlighting a location-specific tissue susceptibility to disease. Different responses of VSMC subtypes to stimuli of growth factors or cytokines likely underlie different disease susceptibility in vessel beds of different locations. Angiotensin II was found to promote proliferation of VSMCs isolated from ascending aorta but not from other aorta regions, whereas platelet-derived growth factor–BB (PDGF-BB) promoted proliferation in VSMCs from all aortic regions (*17*). In addition, transforming growth factor–β1 (TGF-β1) preferentially stimulated the growth and proliferation of neural crest–derived VSMCs over mesoderm-derived VSMCs via c-myb (*48*), and the former resulted in marked elevation of α1 collagen and the latter had no effect on the α1 collagen gene expression (*48*). On the basis of these observations, it is not surprising to see a selective susceptibility of the cerebral VSMCs to the *NOTCH3* mutations in CADASIL. Our finding is in line with a pathological report on a postmortem patient with CADASIL where minimal changes were observed in liver, spleen, heart, and lung whereas significant VSMC disruptions were observed in all small arterioles and capillaries in brain samples (*38*).

It is worth noting that what we identified was the vulnerability of the brain VSMCs to CADASIL *NOTCH3* variants on key functionalities of the mutant VSMCs, i.e., proliferation, migration, cell-matrix interaction, and contraction. This does not exclude the possible pathological changes of peripheral VSMCs via alternative mechanisms or in a later stage. This also should not override the mechanism underlying systemic GOM deposition and Notch3 accumulation that is largely attribute to the conformational change of mutant NOTCH3 proteins due to cysteine alterations (*53*). Since our experiments were mostly conducted on 2D monolayer cell cultures that do not favor GOM or NOTCH3 accumulation, the phenotypes we observed are likely intrinsic changes in mutant VSMCs. This underscores the advantage of using human iPSC model over in vivo models in this context to pinpoint earlier and cell-autonomous pathologies. It is therefore reasonable to speculate that the functional changes in isolated peripheral VSMCs from patients with CADASIL or animal models reported in literature may be largely due to systemic protein accumulations. It would be interesting to clarify this as there is still no clear evidence on whether GOM or NOTCH3 accumulation is a primary driver of CADASIL pathology or secondary effect during disease development. Findings in this study contribute to refining future therapeutic strategies targeting the cerebral vasculature in CADASIL and also highlight the importance of using correct models in studying human disease with iPSCs. Future study is to elucidate the molecular mechanisms by which *NOTCH3* variants specifically alter the function of cerebral VSMCs.

Our results agree with a previous study where increased proliferation was observed on VSMCs from patients with CADASIL (*54*), which together with matrix deposition in small arteries of CADASIL mice (*12*), suggested a phenotype change of VSMCs. Immunostaining of postmortem CADASIL brain tissues identified redistribution of VSMC markers from tunica media to intima (*55*), a hallmark of VSMC phenotypic change contributing to neointimal formation in vascular injury and atherosclerosis (*56*). However, there have not been prior studies specifically addressing the VSMC phenotype in CADASIL. Through analysis of transcriptomic data and functional assays, this study provided comprehensive evidence concluding that CADASIL iVSMCs switched from a contractile to synthetic phenotype. Synthetic VSMCs exhibit a secretory feature, which likely contributes to the ECM accumulation found in CADASIL arteries. However, the accumulated and disorganized ECM may play an even more profound role in driving VSMCs to switch their phenotype. ECM is equally important as growth factors in regulating VSMC phenotypes through binding to their integrin receptors (*36*, *57*). We have found down-regulation of a range of integrin genes expressed in the CADASIL iVSMCs, which likely contributed to the VSMC phenotype alteration. We believe that a vicious cycle is formed where synthetic VSMCs abnormally produce matrix whereas accumulated matrix drives VSMC phenotype changes. Given that phenotype switching is a key feature of atherosclerosis, a major focus of ongoing therapeutic development, highlighting VSMC phenotype switching in CADASIL may open avenues to repurpose treatments designed for more common vascular diseases. Persistent ECM accumulation would lead to progressive wall thickening and fibrosis and luminal narrowing in small arteries, a pathology typically observed in CADASIL small vessels (*9*). Dysregulated integrin signaling, ECM stiffness, and phenotype changes of VSMCs can directly affect VSMC contractility that was demonstrated in this study. Such wider vicious cycle likely accelerates CNS pathologies in CADASIL; therefore, early intervention could benefit patients with CADASIL.

It was observed in early-year CADASIL research that VSMCs from both human patients with CADASIL and mouse models lost anchorage to adjacent cells and ECM, from which it was hypothesized that this might be one of the key events initiating cascades leading to VSMC degeneration in CADASIL (*38*, *58*). In light of the essential role of integrin-ECM engagement in generating prosurvival signals through activation of FAK and the subsequent recruitment of vinculin and the focal adhesion formation (*59*), the significant reduction of FAK, pFAK, and integrins as well as disorganized vinculin in CADASIL iVSMCs found in our study provided molecular basis supporting the hypothesis. GSEA-enriched “adherence junctions” and “cell junction organisation” as well as TEM imaging further support the loss of cell-cell interactions. Abnormal cell-matrix and cell-cell adhesions usually lead to anoikis, a type of apoptosis induced by cell detachment (*41*, *59*, *60*). We observed a significant level of cell death (Fig. 5H) and increased apoptosis in our previous finding (*25*). Thus, the CADASIL iPSCs represent a useful human model for future drug discovery aiming to reverse VSMC survival in this condition.

We provided multiple lines of evidence demonstrating a significant impairment of NO-sGC-cGMP-PKG signaling pathway in CADASIL iVSMCs (Fig. 6). We observed a reduction in NO levels in CADASIL VSMCs, reiterating the role of NO-sGC-cGMP-PKG signaling pathway in VSMCs (*61*) in the absence of ECs that usually supply NO to VSMCs for vessel dilatation. In ECs, eNOS is activated via Akt-mediated phosphorylation. In CADASIL iVSMCs, we identified significantly reduced levels of both Akt and pAkt, which likely contributes to the observed NO deficiency in the mutant cells. In addition, eNOS translocation to the Golgi apparatus is for its palmitoylation, a posttranslational modification required for its recycling back to the plasma membrane caveolae where it can be reactivated by Akt (*46*). The abnormal subcellular localization of eNOS (Fig. 6H) may impair its activation, in addition to reduced eNOS levels, in CADASIL iVSMCs. Consequently, the NO receptor, sGC subunits encoded by *GUCY1A1* and *GUCY1B1*, were down-regulated at both the transcript and protein levels. Moreover, downstream effectors of the cGMP signaling, PKG and VASP, were also reduced. The decreased expression of *GUCY1A1* and *GUCY1B1* may also involve alternative mechanisms. It has previously reported an RBP-Jκ binding site on the *GUCY1A3* promoter region in the developing heart (*62*), linking the canonical Notch signaling to the regulation of sGC. Therefore, the reduced activity of mutant NOTCH3 (fig. S9), although mild, may partially contribute to the down-regulation of *GUCY1A3* in CADASIL VSMCs. Our finding is in line with the report by Neves *et al.* (*63*), where cGMP was reduced in VSMCs from CADASIL mice and patients with CADASIL, although it was caused by oxidation of sGCβ1.

To target the damaged NO-sGC-cGMP signaling pathway, we demonstrated that both NO donor SNAP and PDE5 inhibitor sildenafil could effectively rescue the expression of contractile markers, the abnormal proliferation and angiogenesis, and cell death in the CADASIL iVSMCs. PDE5 specifically targets cellular cGMP for its degradation (*47*). Sildenafil is an FDA-approved medicine primarily for the treatment of erectile dysfunction by augmenting the NO-sGC-cGMP signaling. Although there are controversial findings about the effect of NO on VSMC proliferation, migration, and phenotype switching (*43*), general understanding supports a protective role of NO on VSMC behavior and functions (*42*, *44*, *64*). We previously identified reduced levels of vascular endothelial growth factor (VEGF) in CADASIL iVSMCs, which contributed to impaired angiogenesis (*25*). Several studies have shown that NO induces VEGF synthesis in VSMCs (*65*) and that endogenous NO from ECs promotes angiogenesis (*66*). Moreover, sildenafil has been reported to enhance angiogenesis (*47*, *67*, *68*) and up-regulate VEGF expression (*69*). These findings collectively support our observations. It is possible that the treatment effects by SNAP or sildenafil reflect general cytoprotective actions through the NO-cGMP pathway. However, given that the isoCtrl cells have identical genetic background to the patient iPSCs except for the *NOTCH3* mutation, all the treatments had reversed the disease phenotypes that we have tested to the level of isoCtrls, except for the angiogenesis by SNAP. This suggests that the treatments likely rescued VSMC defects caused by the genetic variants rather than general cytoprotective effects. Future single-cell study may provide more insights into the exact mechanisms of the treatments, especially on 3D models.

To date, accumulating evidence has demonstrated beneficial effects of PDE5 inhibitors in the treatment of a range of common cardiovascular conditions (*47*); a most recent clinical trial using sildenafil to target endothelial functions on patients with sporadic SVD revealed increased cerebrovascular reactivity and perfusion (*70*). Our study added additional mechanisms by which sildenafil improves VSMC function in CADASIL in the absence of ECs. It has been found that PDE5 exists not only in vascular cells but also in human neurons (*66*), which provide a viable target for neurologic disease. An interest study based on a retrospective case-control pharmacoepidemiologic analyses of insurance claims data that included 7.23 million individuals revealed that sildenafil usage was associated with a 69% reduction of Alzheimer’s disease risk (*71*). The beneficial roles of PDE5 inhibition on both vascular and neurologic functions make it a good candidate for treating VaD. Therefore, our results represent a potentially viable therapy for CADASIL.

The work has limitations. Firstly, we acknowledge that iPSC-derived VSMCs were studied in static 2D cultures, without exposure to shear stress, 3D vascular architecture, or coculture with ECs. The absence of these microenvironmental cues may influence VSMC phenotype and weakens extrapolation to cerebrovascular selectivity in vivo*.* Moving forward, it will be beneficial to use 3D models like vascular or vascularized brain organoids derived from patient-specific iPSCs in future work. Secondly, we did not use in vivo models to verify the findings. However, given the species difference between human and animals, it is unlikely that the animal model would truly phenocopy the disease pathophysiology seen in patients with CADASIL. Thirdly, in the PCA plot of the RNA-seq data (Fig. 2A), the controls clustered tightly, but the two lines of patients with CADASIL were separated along PC2, which indicate interpatient heterogeneity, likely attributing to the mutation types and modulation factors such as iPSC line-specific responses to mutations, which warrant further study.

Recently, the FDA passed the Modernization Act 2.0, which eliminated the mandate requirement for animal testing and allowed human cell models to be used in preclinical drug development. This not only reduces the reliance on animal experimentation but also greatly encourages the use of iPSC models in human disease research. Our study demonstrated a robust iPSC CADASIL model, which can be used to further investigate disease mechanisms and facilitate drug development, ultimately benefiting patients.

## MATERIALS AND METHODS

### Cell culture

The human iPSC lines derived from two patients with CADASIL carrying the *NOTCH3* heterozygous variants R153C and C224Y and healthy controls, respectively, were reported in our previous studies with local ethical approval (REC reference no. 12/NW/0533) (*25*, *26*). IsoCtrl lines (R153C_isoCtrl and C224Y_isoCtrl) were created using CRISPR-Cas9 gene editing. IPSCs were maintained in Essential 8 medium (Gibco; A1517001) in a CO_2_ incubator at 37°C. For passaging, subconfluent iPSCs were dissociated using 0.5 mM EDTA (Invitrogen; AM9260G) in phosphate-buffered saline (PBS) at 37°C for 5 to 6 min. Cells were gently rinsed by pipetting in 1 ml of Essential 8 medium, yielding small aggregates that were seeded onto vitronectin (VTN; 0.005 mg/ml; Gibco; A14700)–coated plates at a density of 2.5 × 10^3^ to 3 × 10^3^ cells/cm^2^ in Essential 8 medium supplemented with 10 μM Y-27632 (Tocris; 1254). After 24 hours, the medium was replaced with fresh Essential 8 medium without Y-27632.

### Lineage-specific VSMC differentiation from iPSCs

Differentiation of VSMCs from iPSCs was performed according to published methods (*28*, *72*) with minor modifications. IPSCs with passage numbers of p25 to p35 were used in the study. The same passage numbers were used when comparing results from the mutant and control samples.

#### Neural crest cell differentiation

To generate neural crest progenitor cells, iPSCs were cultured on VTN-coated plates in chemically defined Essential 6 medium (Gibco; A1516401) with fibroblast growth factor 2 (FGF2) (10 ng/ml) (PeproTech; 100-18B) and 10 μM SB431542 (Tocris; 1614). Cells were first passaged at day 5 and subsequently upon reaching confluence, with continued supplementation of FGF2 and SB431542, up to passage 12.

#### Mesodermal progenitor cell differentiation

To induce early mesoderm, human iPSCs were cultured in Essential 6 medium supplemented with FGF2 (20 ng/ml), 10 μM LY294002 (Selleckchem; S1105), and bone morphogenetic protein 4 (BMP4) (25 ng/ml) (PeproTech; 120-05ET) for 48 hours. Subsequent mesodermal subtype specification was directed over the following 4 days. Lateral plate mesoderm (LPM) differentiation was achieved by continued exposure to FGF2 (20 ng/ml) and BMP4 (50 ng/ml), while paraxial mesoderm (PM) differentiation was promoted using FGF2 (20 ng/ml) and 10 μM LY294002.

#### VSMC differentiation from neural crest cells and mesodermal progenitors

Following the generation of intermediate populations, cells were trypsinized and cultured in SMC differentiation medium composed of Essential 6 supplemented with PDGF-BB (10 ng/ml) (PeproTech; 100-14B) and TGF-β1 (2 ng/ml) (PeproTech; 100-21) for a minimum of 12 days. Neural crest–derived VSMCs (NC-SMCs) reached maturity by day 18, while LPM- and PM-derived SMCs matured by day 16. These mature iPSC-derived lineage-specific VSMCs were subsequently maintained in Smooth Muscle Cell Growth Medium 2 (SMCG2; PromoCell; C-22062) for long-term culture, up to five passages.

### Cell proliferation analysis

Cell proliferation was evaluated by Ki67 immunostaining assay. Mature iPSC-VSMCs were seeded on VTN-coated 24-well plates at day 16 for NC-SMCs and day 14 for LPM- and PM-SMCs. After a 48-hour interval, cells were fixed with 4% paraformaldehyde (PFA; Thermo Fisher Scientific; J61899.AK) and subjected to immunostaining for the proliferation marker Ki67 (Abcam; ab15580). The percentage of Ki67-positive cells was quantified using ImageJ software, based on analysis of at least six randomly selected fields per condition in each experiment. The experiment was independently repeated from three separate rounds of differentiation.

### Cell migration assay

Cell migration was assessed by the wound healing assay using the IncuCyte live-cell imaging system (Sartorius; Essen Bioscience). Mature iPSC-derived VSMCs (4 × 10^4^ cells per well) were seeded onto a VTN-coated 96-well ImageLock tissue culture plate (Essen Bioscience) and incubated at 37°C with 5% CO_2_ 24 hours to allow cell attachment and confluence. Wounds were introduced by 96-well WoundMaker (Essen Bioscience), followed by two gentle PBS washes to remove detached cells. Wound closure was monitored in real time by capturing images at 2-hour intervals. Migration was quantified on the basis of changes in wound confluence over time using the IncuCyte analysis software (Essen BioScience). Each condition was tested in technical duplicate, and the experiment was independently repeated from three separate differentiations.

### Collagen I contraction assay

Mature iPSC-derived VSMCs (1 × 10^5^ cells per well) were resuspended in 150 μl of PureCol collagen solution (Advanced Biomatrix; 5005) per well. The collagen-cell suspension was dispensed into 48-well tissue culture plates and allowed to polymerize for an hour at 37°C. After polymerization, the gels were mechanically released by gently running a sterile spatula around the well perimeter, followed by a brief flush with medium to ensure complete detachment. Gel contraction was monitored, and images were captured at 48 hours using a ChemiDoc XRS imaging system (Bio-Rad). The area of each gel was quantified using ImageJ software to assess contraction. Three independent experiments from separate rounds of differentiation were carried out, each performed in triplicate.

### Measurement of intracellular NO levels

Intracellular NO levels in NC-SMCs derived from patients with CADASIL and isoCtrls were measured using the fluorescent probe DAF-FM diacetate (Invitrogen; D23844). Mature NC-SMCs were incubated with 10 μM DAF-FM diacetate in cell culture medium for 30 min at 37°. Following probe loading, cells were washed with PBS and incubated in fresh medium for another 30 min to allow complete intracellular de-esterification. Fluorescence was visualized using an EVOS M3000 microscope with 488-nm excitation. Quantification of fluorescence intensity was performed using ImageJ, with integrated density measurements quantified using ImageJ. Images were acquired from five randomly selected fields per condition, and experiments were conducted in triplicate using VSMCs obtained from three independent differentiations.

### Cell viability assay

Cell viability was assessed using the LIVE/DEAD Cell Imaging Kit (488/570; Invitrogen; R37601). IPSC-derived VSMCs were cultured in a 24-well plate for 48 hours at 37°C in a 5% incubator to allow recovery. Cells were stained according to the manufacturer’s instructions, together with counterstaining Hoechst 33342 (300 ng/ml) to label the nuclei. Fluorescent images were acquired using the EVOS M3000 imaging system (Thermo Fisher Scientific) with excitation at 488 nm (live cells) and 570 nm (dead cells). Cell viability was calculated as the ratio of live cells to total cells (live + dead) based on Hoechst-positive nuclei. For each condition, five random fields were imaged per well, and the experiment was independently repeated three times using VSMCs derived from three separate rounds of differentiation.

### Angiogenesis assay

The angiogenesis was assessed using Matrigel-based endothelial tube formation assay. Growth factor–reduced Matrigel (Corning; 354230) was thawed on ice, dispensed (50 μl per well) into prechilled 96-well plates, and incubated overnight at 37°C in a 5% incubator to allow polymerization. A coculture of 1.5 × 10^4^ human umbilical cord ECs and 0.75 × 10^4^ iPSC-derived VSMCs was plated onto the thin layer of Matrigel in Essential 6 medium supplemented with VEGF-165 (5 ng/ml) (PeproTech; 100-20) and FGF2 (2 ng/ml). Cultures were maintained at 37°C in a humidified 5% CO_2_ incubator to promote capillary-like network formation. Tube-like structures were captured at 1, 4, 7, 10, 13, 24, and 48 hours using bright-field microscopy (EVOS; Thermo Fisher Scientific). Quantification of angiogenic parameters, including the number of nodes, total branching length, and total branches length, was performed using ImageJ with the “Angiogenesis Analyser” plugin. Three independent experiments from separate rounds of differentiation were carried out, each performed in duplicate.

### Generation of spheroids

Size-matched spheroids were generated using iPSC-derived VSMCs on differentiation day 18 using the hanging drop method. Briefly, cells were dissociated with trypsin-EDTA, centrifuged at 300*g* for 5 min, resuspended, and counted using an automated cell counter. Approximately 1 × 10^5^ cells in 20 μl of SMCG2 medium supplemented with 4% poly(vinyl alcohol) (Sigma-Aldrich; 341584) were seeded per drop. After 24 hours, spheroids were collected and transferred to low-attachment 96-well plates. The SMCG2 medium was first replaced at 24 hours and subsequently every 48 hours for the duration of the culture.

### 3D migration assay

Spheroids of iPSC-derived VSMCs were cultured for 24 hours and subsequently embedded in collagen I. The collagen solution was prepared by mixing collagen I matrices (Advanced Biomatrix; 5005) with cell culture medium at a 2:1 ratio, and ~300 μl of the mixture was added per well in a 48-well plate. After a 5-min settling period, four to five spheroids were randomly placed into each well. Plates were incubated at 37°C with 5% CO_2_ for 1 hour to allow gel polymerization. To inhibit proliferation and focus on cell migration, spheroids were treated with mitomycin C (25 μg/ml) (Stemcell Technology; 73274) for 20 min, followed by replacement with fresh culture medium. Bright-field images were acquired daily to monitor spheroid outgrowth. Migration distances were quantified using ImageJ by converting images to 8-bit format with the “Mask” function to enhance visualization and measurement. Each data point in the violin plot represents an individual spheroid. Three independent experiments from separate rounds of differentiation were carried out.

### Transmission electron microscope protocol for spheroids

Spheroids were fixed immediately in a solution of 2.5% glutaraldehyde and 4% formaldehyde in 0.1 M Hepes buffer (pH 7.2) for 2 hours. Samples were then rinsed in distilled water. Samples were then postfixed in a solution of 1.0% osmium tetroxide and 1.5% potassium ferrocyanide in 0.1 M cacodylate buffer for 2 hours and washed with H_2_O three times to remove osmium tetroxide residuals. Specimens underwent incubation in thiocarbohydrazide (TCH) for 60 min at room temperature (RT) followed by five-times washing with distilled water, until any formed TCH crystals were dissolved. Then, these spheroids were incubated in 1% osmium tetroxide in distilled water for 1 hour before incubated in 1% uranyl acetate for another an hour. These specimens underwent dehydration steps in ascending ethanol series (30, 50, 70, 90, and 100%, v/v × 3). Specimens were infiltrated in graded TAAB 812 Hard in acetone at RT overnight. Last, samples were embedded in free 100% TAAB 812 Hard in a labeled mold and put in a stove at 60°C for 48 hours. Ultrathin sections for TEM observations were cut using an ultramicrotome (Leica EM UC6, Vienna, Austria). Ultrathin sections were collected on 100-mesh copper grids (Assing, Rome, Italy) stained with Uranyless solution and lead citrate 3% solution (Electron Microscopy Science, 1560 Industry Road, Hatfield, PA, USA). Imaging was performed with a transmission electron microscope (Carl Zeiss EM10, Thornwood, NY, USA) set with an accelerating voltage of 60 kV. Images were acquired with a charge-coupled device (CCD) digital camera (AMT CCD, Deben UK Ltd., Suffolk, UK).

### Luciferase reporter assay

The activation of NOTCH signaling pathway was detected by Dual-Glo Luciferase Assay System (Promega; E2920) as we previously described (*14*). IPSC-derived VSMCs were plated into a 12-well plate and transfected with 4xCSL-luciferase reporter vector (Addgene plasmid 41726, 1 μg) and *Renilla* luciferase (0.5 μg) using Lipofectamine LTX reagent with Plus reagent (Thermo Fisher Scientific, 15338030) following the manufacturer’s instructions. After 24 hours of transfection, the activity of firefly luciferase and *Renilla* luciferase was quantified in a 96-well plate by a luminometer using the Dual-Glo luciferase kit according to the instructions provided by the manufacturer.

### Real-time quantitative PCR

Cells were harvested from six-well culture plates, and total RNA was extracted using an RNeasy Mini kit (Qiagen; 74104). Then, cDNA was synthesized using the high-capacity RNA-to-cDNA kit (Invitrogen; 4388950). Quantitative real-time PCR was performed using the QuantStudio 6 Real-Time system (Thermo Fisher Scientific) using Power SYBR Green PCR Master Mix (Applied Biosystems; 4367659). Gene expression levels were normalized to *GAPDH* as the housekeeping gene, and relative quantification was calculated using the Pfaffl method. Each sample was test in technical triplicate, and the experiment was repeated independently three times using iPSC-derived VSMCs from three separate rounds of differentiation. The primer sequences and efficiency are shown in table S1.

### Western blotting

Cells were lysed with radioimmunoprecipitation assay buffer (Sigma-Aldrich; R0278) supplemented with 100× EDTA-free Halt Protease Inhibitor Cocktail (Thermo Fisher Scientific, 87785), and lysates were centrifuged at 16,200*g* for 20 min at 4°C. The supernatant was collected, and protein concentration was determined using the Pierce BCA protein assay kit (Thermo Fisher Scientific, 23225). For each sample, 20 μg of total protein was mixed with NuPAGE lithium dodecyl sulfate sample buffer and β-mercaptoethanol and then boiled at 95°C for 5 min. Proteins were resolved on 12% or 4 to 20% TGX stain-free precast gels (Bio-Rad) and transferred onto nitrocellulose membranes. Membranes were blocked in TBS containing 0.1% (v/v) Tween 20 (TBST) supplemented with 5% (w/v) slim milk powder and 2% (w/v) bovine serum albumin (BSA) for an hour at RT, followed by incubation with primary antibodies overnight at 4°C. After washing with TBST, blots were incubated with proper horseradish peroxidase–conjugated secondary antibodies for 2 hours at RT. Signal detection was performed using SuperSignal West Pico or Femto Chemiluminescent Substrate (Thermo Fisher Scientific), and blots were imaged using autoradiography. Where indicated, band intensities were quantified by densitometry using Image Lab software (Bio-Rad).

### IF on 3D spheroids

IF staining was performed to determine NOTCH3 (R&D; AF1308) and collagen IV (Thermo Fisher Scientific; PA5-104508) proteins in 3D VSMC spheroids. Spheroids were cultured in SMCG2 medium for an additional 7 days to promote ECM formation. Subsequently, spheroids were collected into 1.5-ml Eppendorf tubes, washed with PBS, and fixed in 4% PFA for 2 hours at 4°C. Fixed spheroids were permeabilized with 0.5% (v/v) Triton X-100 in PBS for 4 hours at RT, followed by blocking overnight in IF buffer containing 10% donkey serum, 2% (w/v) BSA, 0.2% (v/v) Triton X-100, and 0.05% (v/v) Tween 20 in PBS. Primary anti-human antibodies NOTCH3 and collagen IV were diluted in fresh IF buffer and incubated the spheroids for 3 days at RT. After primary incubation, spheroids were washed three times with IF buffer and incubated for 24 hours at RT with species-specific secondary antibodies (anti-goat Alexa Fluor 488 and anti-rabbit Alexa Fluor 647) and Hoechst 33342 (1:50,000 dilution) diluted in IF buffer. Following three additional washes, spheroids were mounted in 0.5-mm-deep iSpacer chambers filled with Rapiclear 1.49 (Sunjin Lab Co.). Spheroids were imaged with a Leica SP8 confocal microscope under a 10× objective. Z-stacks were acquired and processed using ImageJ to generate either 2D maximum intensity projections or 3D reconstructions.

### IF on monolayer iPSC-derived VSMCs

IPSC-derived VSMCs were cultured on VTN-coated coverslips or 24-well plates and fixed with 4% PFA for 15 min at RT. Following fixation, cells were permeabilized with 0.5% Triton X-100 in PBS for 20 min and blocked in IF buffer an hour at RT. Cells were then incubated with primary antibodies: anti–α-SMA antibody (1:250, Abcam, ab7817), anti-calponin (1:250, Abcam, ab46794), anti-transgelin (1:500, Abcam, ab14106), anti-vinculin (1:500, Sigma-Aldrich, V9131), anti-iNOS (1:200; Novus, NB300), anti-GM130 (1:200; Novus; AF8199-SP), and anti-eNOS (1:300; Proteintech; 27120-1-AP) diluted in IF buffer overnight at RT. After washing, appropriate Alexa Fluor–conjugated secondary antibodies were applied for 2 hours at RT in darkness. Nuclei were counterstained with 4′,6-diamidino-2-phenylindole (DAPI). Coverslips were mounted using Fluoromount-G mounting medium (Invitrogen; 00-4958-02), and images were acquired with a fluorescence or Leica SP8 confocal microscope. Quantification of fluorescence intensity or positive cell percentage was performed using ImageJ.

### F-actin staining and quantification

Immature iPSC-derived VSMCs on differentiation day 12 were placed onto VTN-coated glass coverslips and cultured in SMC differentiation medium. At day 18, cells were fixed with 4% PFA for 15 min at RT, permeabilized with 0.5% Triton X-100 in PBS for 10 min, and blocked in IF buffer for 30 min. For visualization of the cytoskeletal structure, F-actin was stained using Phalloidin-iFluor 488 Reagent (Abcam, ab176753) for 2 hours at RT. Nuclei were counterstained with DAPI. Images were acquired using a Leica SP8 inverted confocal microscope equipped with a 100× oil immersion objective.

Spatial dispersion is used to quantify cytoskeletal organization. We resize the input image to 512 by 512 pixels. Then, the standard Canny edge detection algorithm (*73*) is used to outline the edges of stress fibers. First, a Gaussian filter is applied to smoothing the image while reducing image noise. Afterward, image gradients are computed so that potential edges can be better identified. The detected edges are then processed using a Non-Maximum Suppression technique (*74*) to eliminate false detections, followed by edge screening via a dual-threshold approach of Canny edge detection (*73*). The dual thresholds are determined on the basis of statistical characteristics of image pixels. To be specific, the standard Sobel operator (*75*) is used to calculate horizontal (*x*) and vertical (*y*) gradients ($G_{x}$ and $G_{y}$). The gradient magnitude *G* at each pixel, representing gradient intensity, is computed as

$$G=\sqrt{G_{x}^{2}+G_{y}^{2}}$$

The mean (*M*) and standard deviation (*S*) of the gradient magnitudes are then calculated. The upper threshold ($T_{\text{max}}$) and the lower threshold ($T_{\text{min}}$) are determined as follows, where *T* denotes the reduction ratio

$$T_{\text{max}}=M+2\times S$$

$$T_{\text{min}}=T_{\text{max}}/T$$

Next, the resized image is converted to the counterpart in the hue, saturation, and value (HSV) color space. The center point of the blue region is identified on the basis of a predefined HSV range. For each stress fiber edge, two parameters are computed:

1) The distance ($D^{i}$) from the center of the blue region to the edge

2) The angle ($A^{i}$) between the edge and the horizontal axis (0° < $A^{i}$ < 180°)

For the images containing multiple blue regions, the average distance ($D_{\text{avg}}^{i}$) from each stress fiber edge to all the blue centers is calculated individually. During this process, small blue regions and short stress fiber edges are filtered out.

Last, spatial dispersion (*dsp*) is used to measure the degree of disorder. Each pair ($D_{\text{avg}}^{i}$, $A^{i}$) is treated as a point in the 2D space. The space is divided into *n* subspaces, and the probability *p* of the points falling into each subspace is computed as the ratio of the points in that specific subspace against the total number of the points. The entropy (*etp*) and the normalized spatial dispersion (*dsp*) are derived as follows

etp=sum[−p×log(p)]

$$\text{dsp}=\frac{\text{etp}}{-\text{log}\left(\frac{1}{n}\right)}$$

where *etp* is used to measure the uncertainty of the point distribution within spatial subregions, where *p* represents the proportion of the points in each subregion relative to the total number of the points. *n* denotes the total number of the subregions. By dividing *etp* by the maximum entropy (${\text{log}}_{2}n$), the result is normalized to a scale of [0,1], facilitating the comparisons across different regions.

This code is available at https://github.com/zhaoaite/ActinDetection.

### RNA-seq sample processing, sequencing, and data processing

RNA-seq was performed by the Genomic Technologies Core Facility at the University of Manchester. The quality and integrity of the total RNA samples were assessed using a 4200 TapeStation (Agilent Technologies, Cheadle, UK), and then libraries were generated using the Illumina Stranded mRNA Prep Ligation kit (Illumina Inc., Cambridge, UK) according to the manufacturer’s protocol. Briefly, total RNA (typically 0.025 to 1 μg) was used as input material, from which polyadenylated mRNA was purified using poly-T, oligo-attached, magnetic beads. Next, the mRNA was fragmented under elevated temperature and then reverse transcribed into first-strand cDNA using random hexamer primers and in the presence of actinomycin D (thus improving strand specificity while mitigating spurious DNA-dependent synthesis). Following removal of the template RNA, second-strand cDNA was then synthesized to yield blunt-ended, double-stranded cDNA fragments. Strand specificity was maintained by the incorporation of deoxyuridine triphosphate in place of deoxythymidine triphosphate to quench the second strand during subsequent amplification. Following a single adenine (A) base addition, adapters with a corresponding, complementary thymine (T) overhang were ligated to the cDNA fragments. Preindex anchors were then ligated to the ends of the double-stranded cDNA fragments to prepare them for dual indexing. A subsequent PCR amplification step was then used to add the index adapter sequences to create the final cDNA library. The adapter indices enabled the multiplexing of the libraries, which were pooled before cluster generation using a cBot instrument. The loaded flow cell was then paired-end sequenced (76 + 76 cycles, plus indices) on an Illumina HiSeq 4000 instrument. Last, the output data were demultiplexed, and BCL-to-Fastq conversion was performed using Illumina’s bcl2fastq software, version 2.20.0.422 (Illumina Inc., San Diego, CA, USA).

For RNA-seq data analysis, the unmapped paired-end sequences from the HiSeq 4000 were assessed by FastQC (www.bioinformatics.babraham.ac.uk/projects/fastqc/, accessed on 15 October 2021). Sequence adapters were removed, and reads were quality trimmed to quality q20 using Trimmomatic_0.36 (PMID: 24695404). The reads were mapped against the reference human (hg38) genome, and counts per gene were calculated using annotation from GENCODE 30 (www.gencodegenes.org/, accessed on 15 October 2021) using STAR_2.5.3a (PMID: 23104886). Downstream normalization, PCA, and differential expression were calculated in DESeq2_1.20.0 using default settings (PMID:25516281). Differentially expressed genes were identified between NC-SMCs derived from patients with CADASIL and controls (isoCtrl and wild-type) using an adjusted *P* < 0.05 and |log_2_(fold change)| > 1.

Results were visualized using heatmaps generated with the pheatmap package (v1.0.12; R Foundation for Statistical Computing, Vienna, Austria). The volcano plots derived from DESeq2 statistical outputs was plotted by https://www.bioinformatics.com.cn (last accessed on 10 December 2024), an online platform for data analysis and visualization. GO and KEGG pathway enrichment analyses, as well as GSEA, were conducted using the clusterProfiler package (v3.16.0; Bioconductor, Boston, MA, USA) to characterize biological processes and signaling pathways enriched among significantly up-regulated or down-regulated genes, and the pictures were plotted by https://www.bioinformatics.com.cn.

### Statistical analysis

All statistical analyses were performed using GraphPad Prism version 10.0 (GraphPad Software, San Diego, CA, USA) and R version 4.4.2 (R Foundation for Statistical Computing, Vienna, Austria). Data are presented as means ± SEM. Shapiro-Wilk test was used to assess the normality of data distributions. For comparison between samples of patients with CADASIL and their respective isoCtrls, unpaired Student’s *t* test was used. One-way analysis of variance (ANOVA) followed by Tukey’s post hoc test was applied for multiple comparisons among the three lineage-specific VSMCs. For experiments involving two independent variables, *NOTCH3* variants and treatment, two-way ANOVA followed by Tukey’s multiple comparisons test was conducted to evaluate group differences. A *P* value of less than 0.05 was considered statistically significant. All experiments were conducted with a minimum of three biological replicates as three independent rounds of differentiation from each iPSC line with two to three technical repeats in each experiment. Detailed sample size and number of replicates are also specified in the corresponding figure legend.
