# Supplementary material for: Selective vulnerability of cerebral vasculature to NOTCH3 variants in small vessel disease and rescue by phosphodiesterase-5 inhibitor
Source: Sci Adv. 2026 Apr 3;12(14):eaeb1134. doi: 10.1126/sciadv.aeb1134 (PMC13048245; doi:10.1126/sciadv.aeb1134)
Supplement: Supplementary file 1 — Figs. S1 to S13 Table S1 Appendix 1 [file sciadv.aeb1134_sm.pdf]

Science Advances
AAAS

Supplementary Materials for

Selective vulnerability of cerebral vasculature to *NOTCH3* variants in small vessel disease and rescue by phosphodiesterase-5 inhibitor

Xiangjun Zhao *et al.*

Corresponding author: Tao Wang, tao.wang@manchester.ac.uk

Sci. Adv. **12**, eaeb1134 (2026)
DOI: 10.1126/sciadv.aeb1134

This PDF file includes:

Figs. S1 to S13
Table S1
Appendix 1

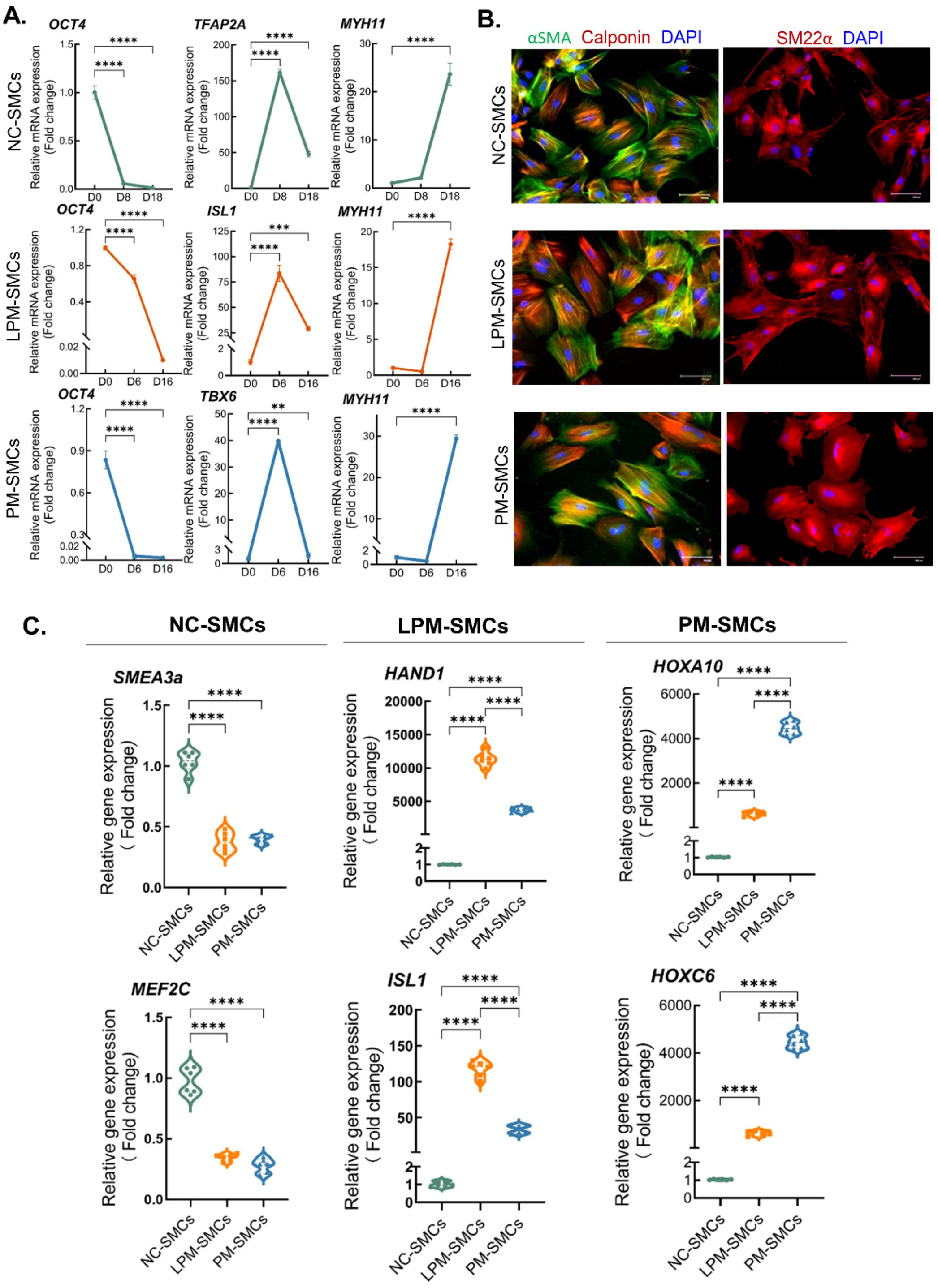

Fig. S1. The verification of lineage-specific iPSC-derived VSMCs. IPSCs were differentiated into VSMCs via neural crest (NC-SMCs), lateral plate mesoderm (LPM-SMCs), and paraxial mesoderm (PM-SMCs) lineages. (**A**) RT-qPCR analysis showing loss of pluripotency marker gene *OCT4*, upregulation of lineage-specific progenitor markers (*TFAP2A*, *ISL1* and *TBX6*), and increased expression of VSMC marker gene *MYH11* during differentiation. (**B**) Immunofluorescence staining of VSMC on day 18 differentiation demonstrating expression of α-SMA (green), calponin (red), and SM22α (red). Nuclei were counterstained with DAPI (blue). Scale bar = 100μm. (**C**) RT-qPCR analysis showing expression of lineage-specific markers: *SEMA3A* and *MEF2C* for NC-SMCs; *ISL1* and *HAND1* for LPM-SMCs; and *HOXA10* and *HOXC6* for PM-SMCs. Data are presented as mean ± SEM. Figures (**A**) and (**B**) were from 3 independent iPSC differentiations (n=3), and Figure (C) were from 6 independent differentiation (n=6). Statistical analysis was performed using one-way ANOVA with Tukey's post hoc test, $^{**}p \leq 0.01$, $^{***}p \leq 0.001$, $^{****}p \leq 0.0001$.

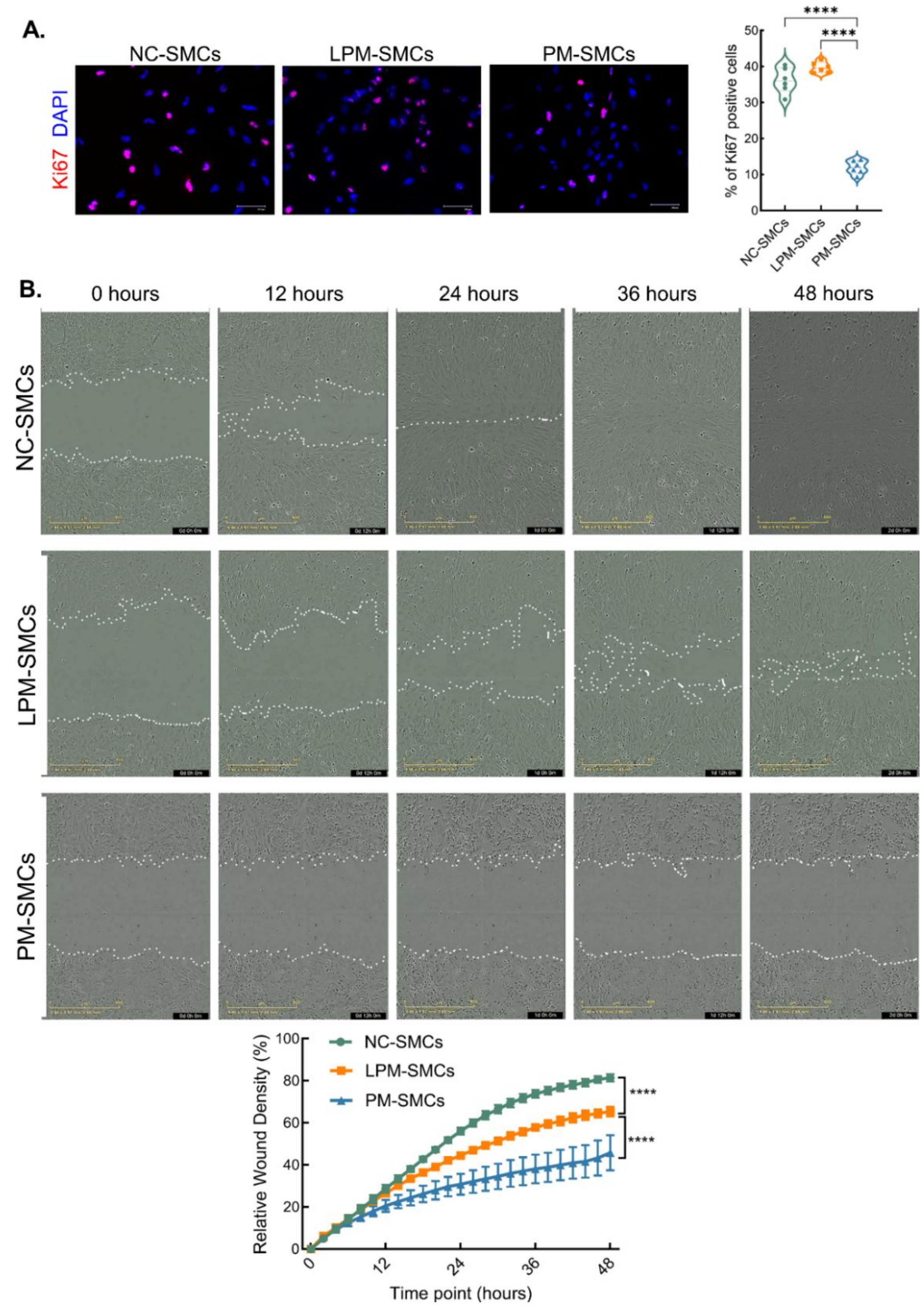

Fig. S2. Proliferation and migration of iPSC derived lineage-specific VSMCs. IPSCs were differentiated into VSMCs via neural crest (NC-SMCs), lateral plate mesoderm (LPM-SMCs) and paraxial mesoderm (PM-SMCs) lineages. (**A**) Cell proliferation was determined by ki67 immunofluorescent staining (left) and quantified (right). Scale bar = 100 µm**.** (**B**) Cell migration was evaluated using IncuCyte live-cell imaging of wound healing assay over 48 hours (top), with quantification shown (bottom). Scale bar = 500 µm. Data are presented as mean ± SEM from at least three independent iPSC differentiations (n = 3). Statistical significance was determined using one-way ANOVA with Tukey's post hoc test in (A), and two-way ANOVA followed by post hoc analysis in (B). ****$p \leq 0.0001$.

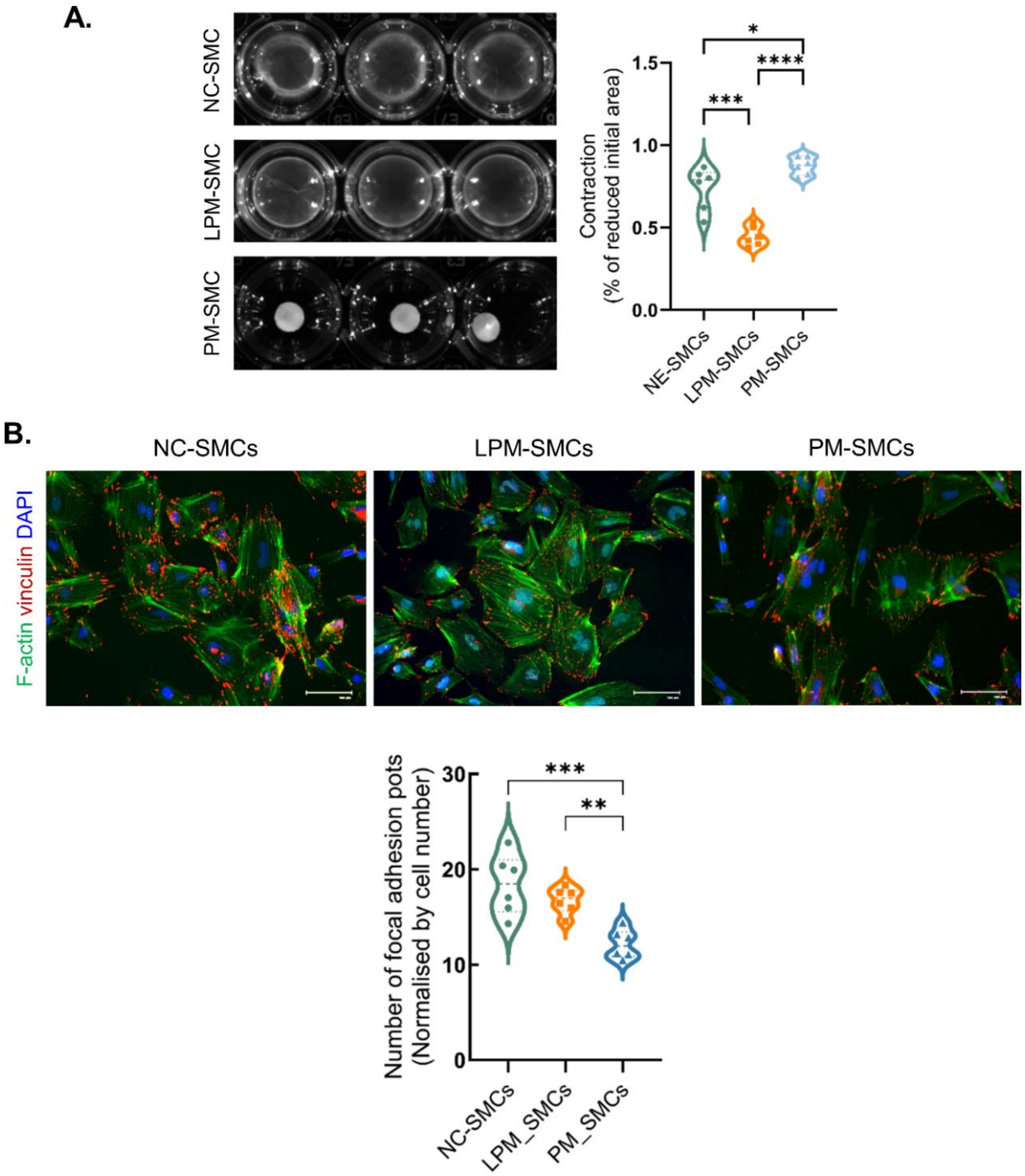

Fig. S3. Contraction and extracellular matrix interaction of lineage-specific VSMCs derived from iPSCs. IPSCs were differentiated into VSMCs via neural crest (NC-SMCs), lateral plate mesoderm (LPM-SMCs) and paraxial mesoderm (PM-SMCs) lineages. (**A**) Cell contractility was assessed using collagen I gel contraction assay (left), with quantification shown (right). (**B**) Focal adhesions were visualised by vinculin immunofluorescent staining (red) and quantified (bottom). F-actin was stained (green). Scale bars = 100 µm. Data are presented as mean ± SEM from at least three independent iPSC differentiations (n = 3). Statistical significance was determined using one-way ANOVA followed by Tukey's post hoc test. $^*p \leq 0.05$, $^{**}p \leq 0.01$, $^{***}p \leq 0.001$.

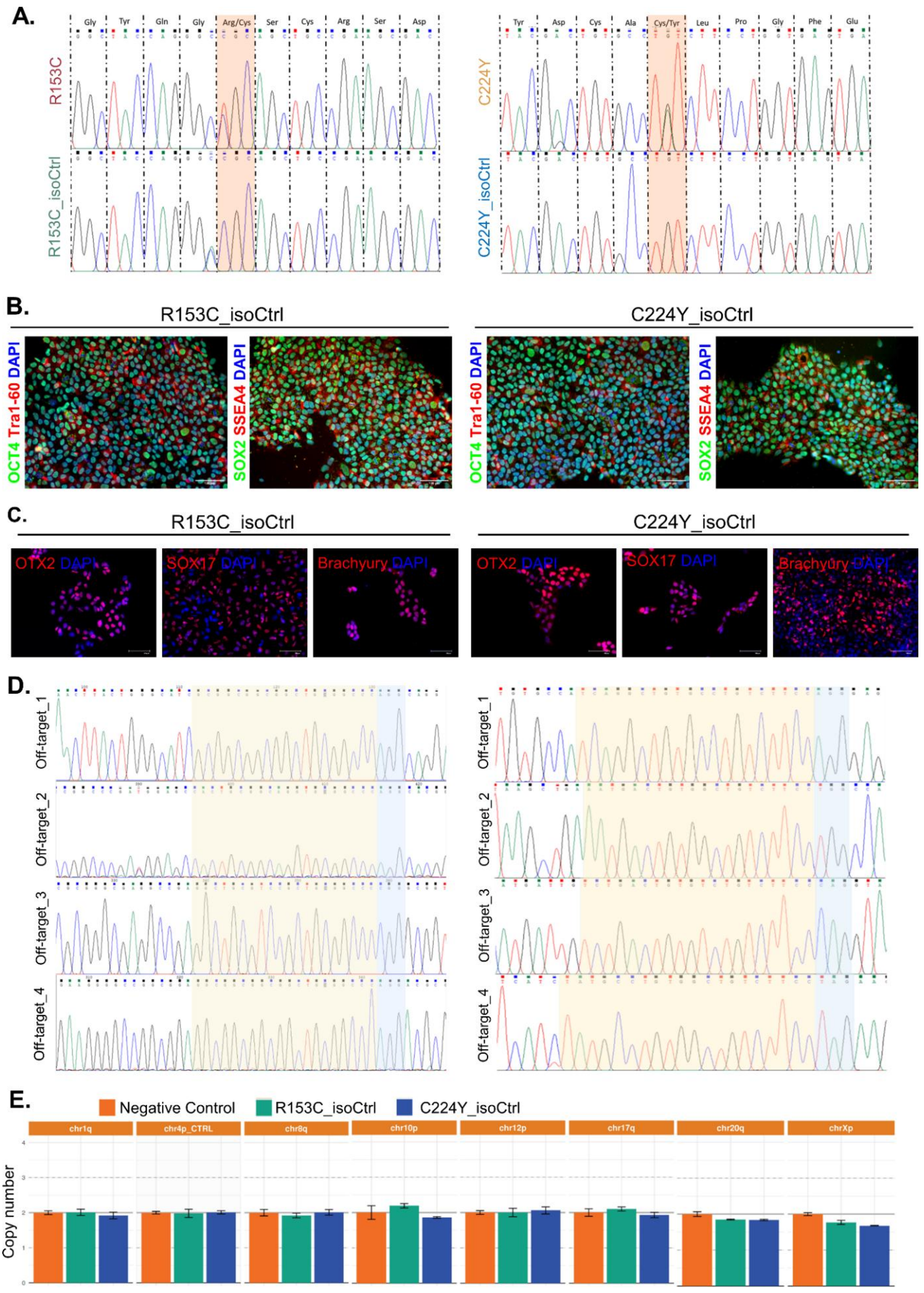

A.
Gly Tyr Gln Gly Arg/Cys Ser Cys Arg Ser Asp
R153C
R153C_isoCtrl
Tyr Asp Cys Ala Cys/Tyr Leu Pro Gly Phe Glu
C224Y
C224Y_isoCtrl
B.
R153C_isoCtrl
C224Y_isoCtrl
OCT4 Tra1-60 DAPI
SOX2 SSEA4 DAPI
C.
R153C_isoCtrl
C224Y_isoCtrl
OTX2 DAPI
SOX17 DAPI
Brachyury DAPI
D.
Off-target_1
Off-target_2
Off-target_3
Off-target_4
E.
Negative Control
R153C_isoCtrl
C224Y_isoCtrl
chr1q
chr4p_CTRL
chr8q
chr10p
chr12p
chr17q
chr20q
chrXp
Copy number
0
1
2
3
4

Fig. S4. Generation and characterisation of isogenic control iPSC lines. IPSC lines carrying CADASIL-associated *NOTCH3* variants R153C and C244Y were edited using CRISPR/Cas9 to generate isogenic control lines (isoCtrls) by correcting the respective mutations. (**A**) Sanger sequencing chromatograms confirming correction of R153C and C224Y variants back to wild type sequence. (**B**) Immunofluorescence staining for pluripotency markers OCT4 (octamer-binding transcription factor 4, green), SOX2 (SRY-box transcription factor 2, green), Tra1-60 (tumor rejection antigen-1-60, red) and SSEA4 (stage-specific embryonic antigen-4, red). Nuclei were counterstained by DAPI (blue). Scale bar = 100 µm. (**C**) Immunofluorescence analysis of tri-lineage differentiation demonstrating expression of ectodermal marker OTX2 (orthodenticle homeobox 2, red), endodermal marker SOX17 (SRY-box transcription factor 17, red), and mesodermal marker Brachyury (T-box transcription factor, red), confirming pluripotency. Nuclei were counterstained by DAPI (blue). Scale bar = 100 µm. (**D**) Sanger sequencing of the four top predicted CRISPR off-target sites showed no detectable off-target editing. (**E**) hPSC Genetic Analysis Kit (StemCell Technologies) confirming normal copy number across eight chromosomal loci commonly affected in human iPSCs during culture.

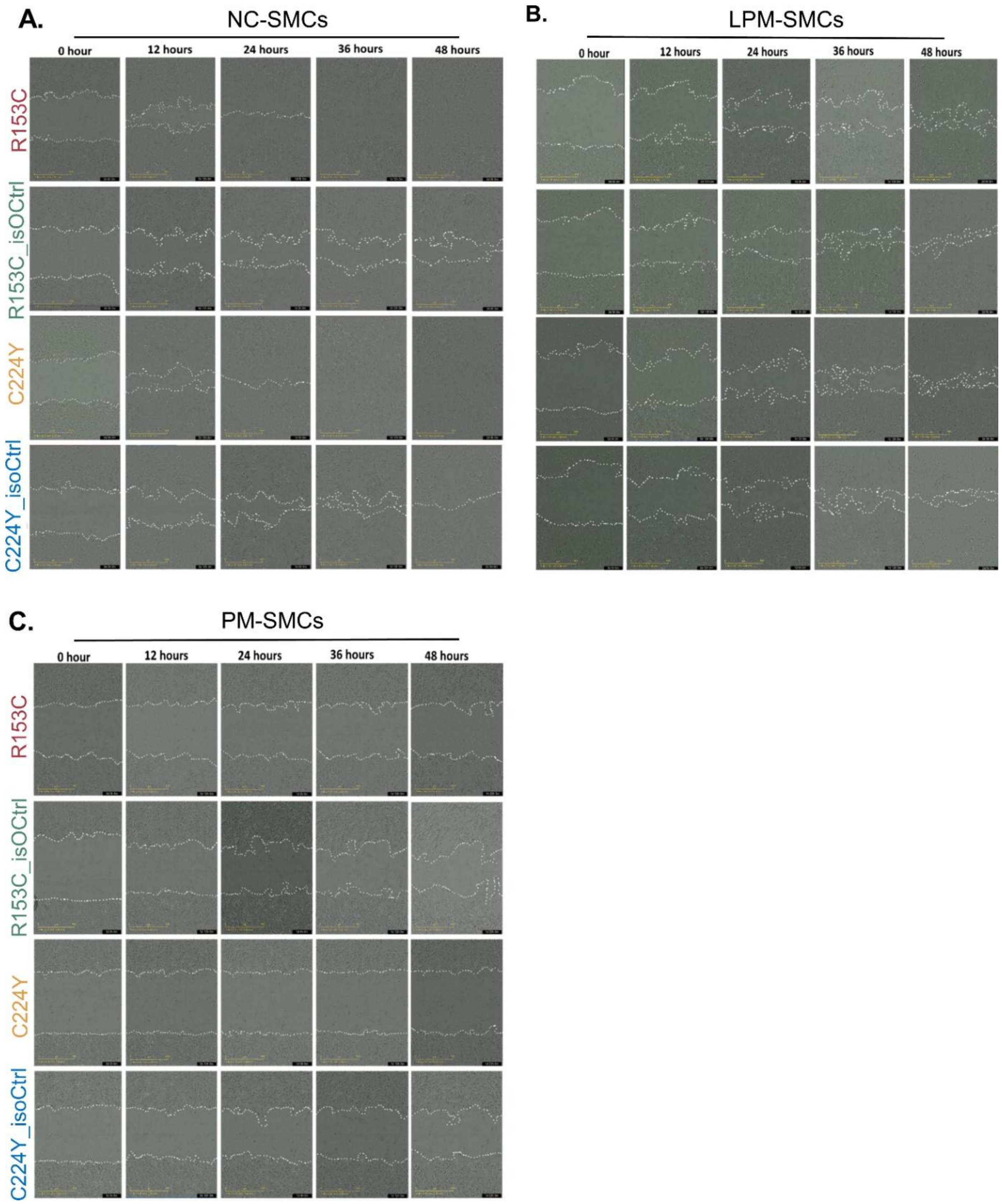

Fig. S5. Migration assay of iPSC-derived lineage-specific VSMCs by wound healing assay. IPSCs from two CADASIL patients (R153C and C224Y) and their isogenic controls (isoCtrl) were differentiated into VSMCs through neural crest (NC-SMCs), lateral plate mesoderm (LPM-SMCs) and paraxial mesoderm (PM-SMCs) lineages. Migratory capacity was measured using live cell imaging in a scratch wound healing assay (IncuCyte). Representative microscope images of migrating VSMCs were acquired at 0, 12, 24, 36 and 48 hours. White lines indicate the leading edge of migrating cells. Scale bar = 600 μm.

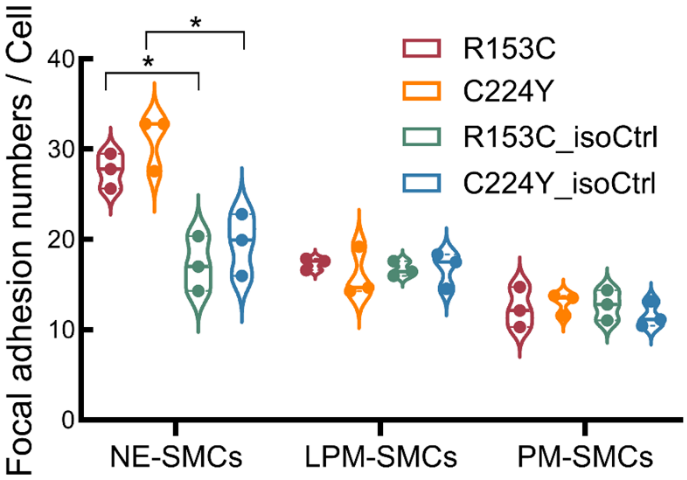

Fig. S6. Quantification of focal adhesions. IPSCs from two CADASIL patients (R153C and C224Y) and their isogenic controls (isoCtrl) were differentiated into VSMCs through neural crest (NC-SMCs), lateral plate mesoderm (LPM-SMCs) and paraxial mesoderm (PM-SMCs) lineages. Immunostaining for vinculin was carried out on the iPSC derived VSMCs to visualize focal adhesions. The number of focal adhesions were quantified. Data are mean ± SEM from three independent iPSC differentiations (n=3). Unpaired Student's t test *p ≤ 0.05.

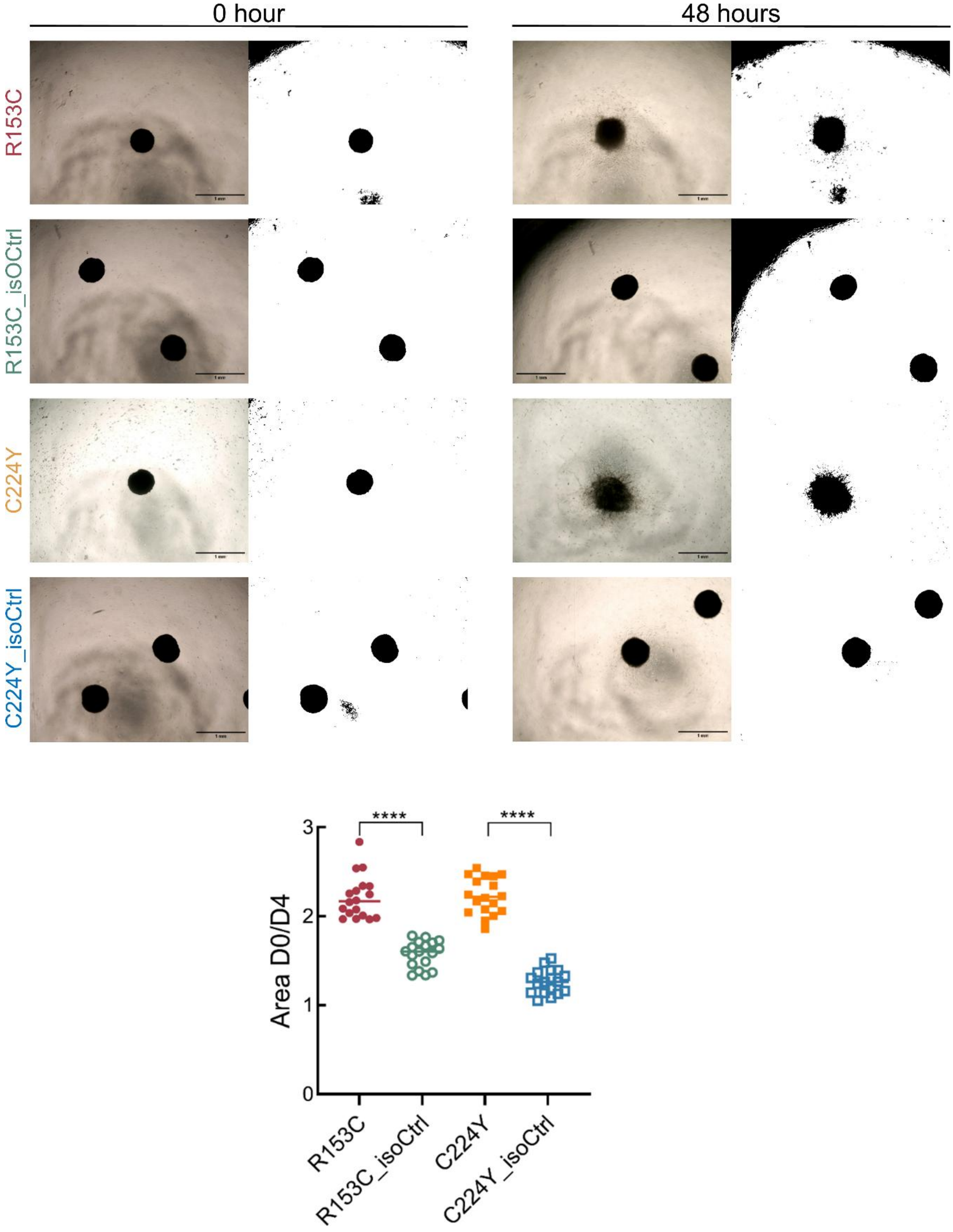

Fig. S7. Migration of iPSC-derived VSMCs assessed by spheroid migration assay. IPSCs from two CADASIL patients (R153C and C224Y) and their isogenic controls (isoCtrls) were differentiated into iVSMCs via neural crest lineage and then cultured as spheroids to assess cell migration. (**A**) Representative light microscopy images of spheroids from CADASIL and isoCtrl lines over 0-48 hours (D0; Day 0; D4: Day4). Scale bar = 1 mm. (**B**) Spheroid outgrowth was quantified by measuring flat surface area using ImageJ. Data are presented as the mean ± SEM of 18 spheroids of each cell line from at least three independent biological replicates (n = 3). Statistical significance was determined using unpaired student's *t-test*. ****$p \leq 0.0001$.

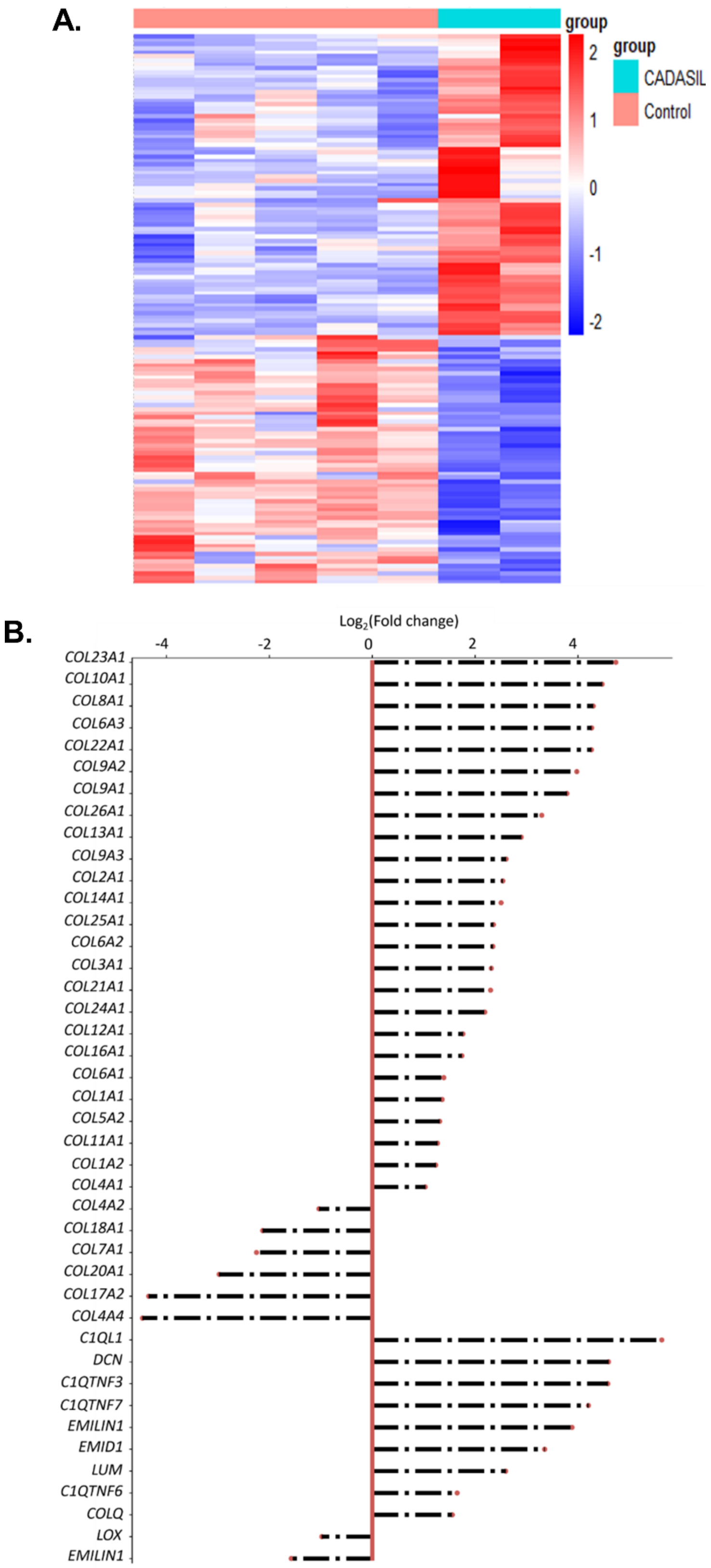

Fig. S8. RNA-seq analysis of extracellular matrix (ECM)-related gene expression in iPSC-derived VSMCs. IPSCs from two CADASIL patients (R135C & C224Y) and respective isogenic controls (isoCtrls) were differentiated into iVSMCs via neural crest lineage and subjected to RNA sequencing. (**A**) Heatmap of differentially expressed genes associated with the Gene Ontology (GO) term "extracellular matrix organization". (**B**) Differential expression of collagen-related genes between CADASIL and isoCtrl iVSMCs.

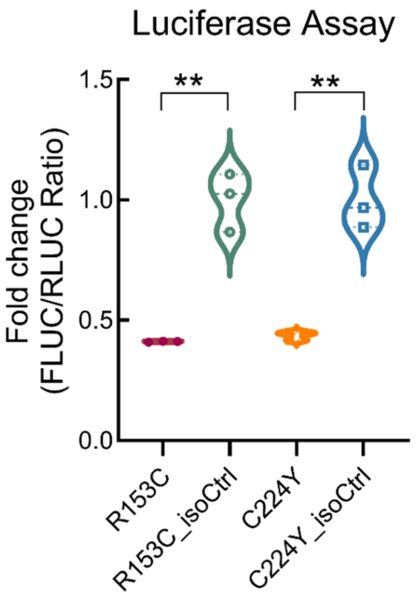

Fig. S9. Notch signalling activity assessed by luciferase reporter assay. IPSCs from two CADASIL patients (R153C and C224Y) and their isogenic controls (isoCtrl) were differentiated into iVSMCs via neural crest lineage. The canonical NOTCH signalling activity was measured using Dual-Glo Luciferase reporter assay. Relative luciferase activities were presented as the ratio of Firefly luciferase (FLUC) to Renilla luciferase (RLUC) (FLUC/RLUC) and normalised to isoCtrl values. Data are represented as mean ± SEM from 3 independent iPSC differentiations (n=3). Unpaired Student's *t* test, $^{**}p \leq 0.01$.

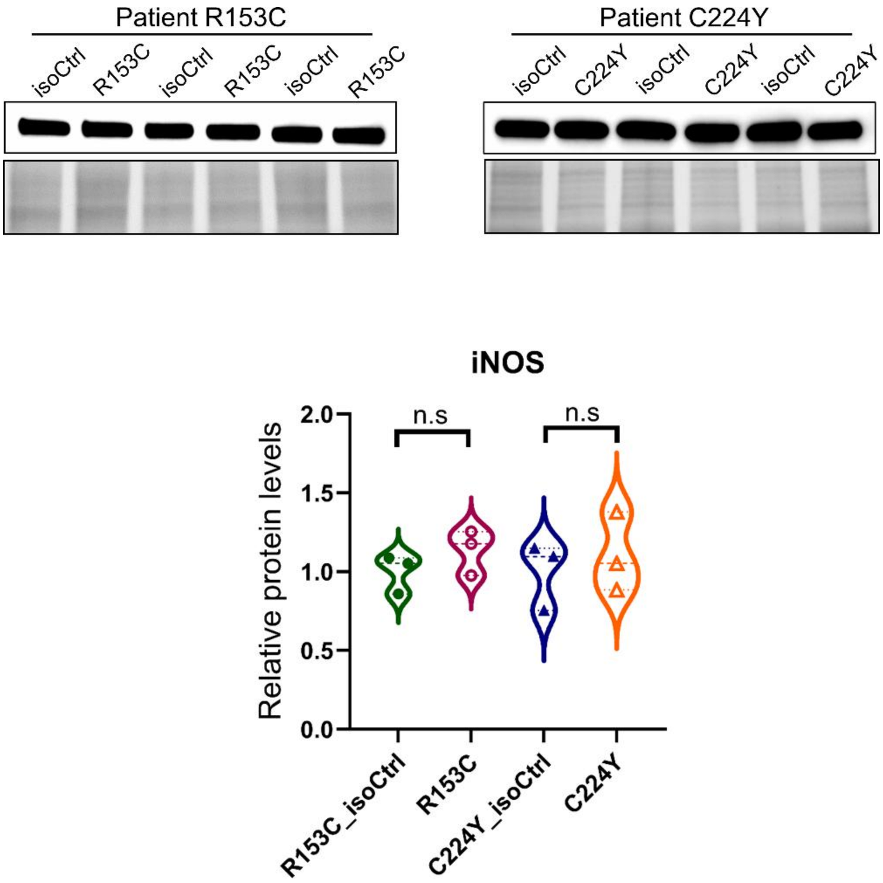

Fig. S10. Quantification of inducible oxide synthase (iNOS) in iPSC-derived CADASIL patient and isoCtrl iVSMCs. IPSCs from two CADASIL patients (R153C and C224Y) and their isogenic controls (isoCtrls) were differentiated into iVSMCs via neural crest lineage. The iVSMCs were subjected to western blotting for iNOS and quantified (lower panel). The protein levels were normalized to total protein loading controls (representative loading areas shown below each blot). Data are mean ± SEM from three independent iPSC differentiations (n=3). Unpaired Student's *t* test, n.s., no significant difference.

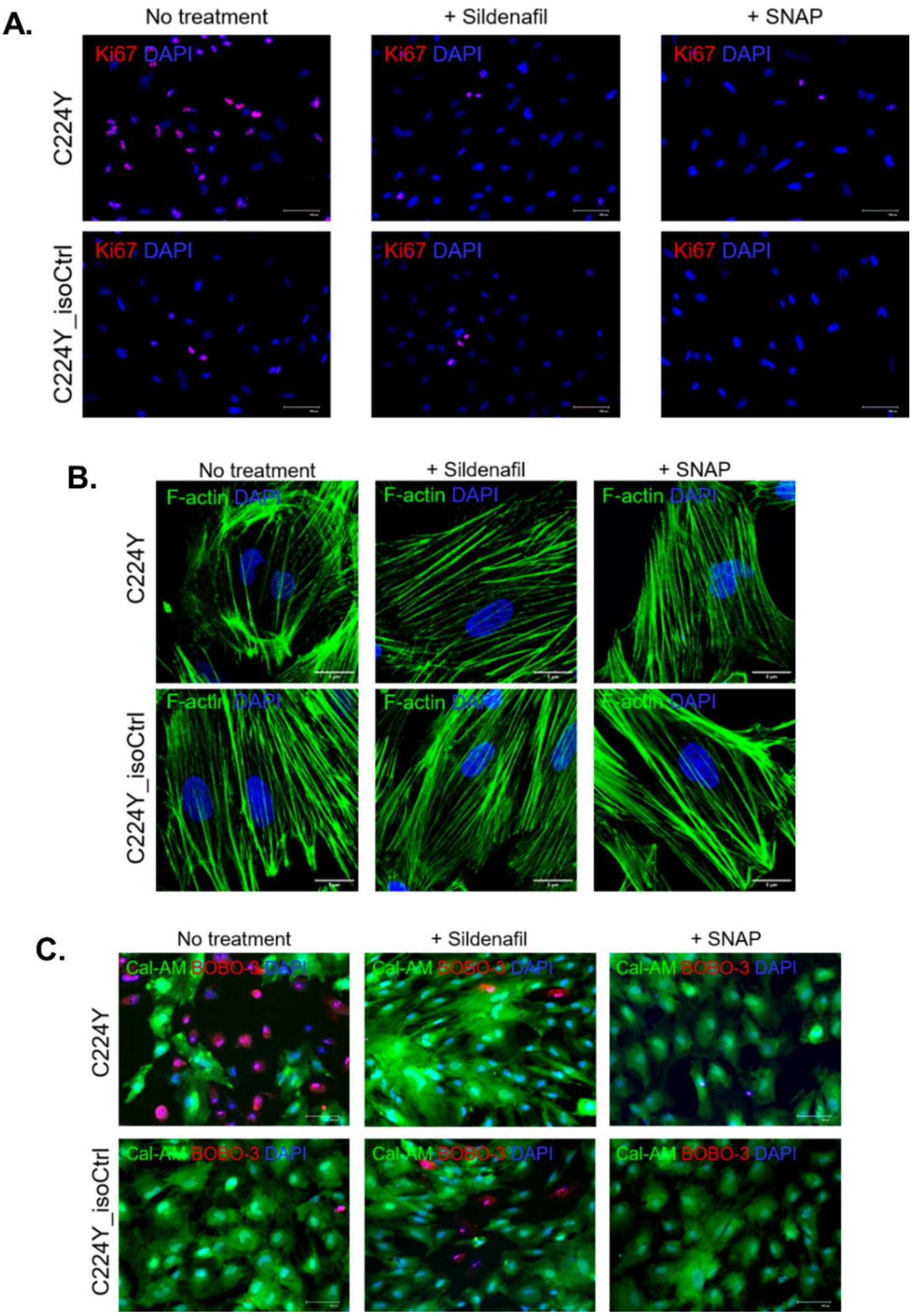

Fig. S11. Rescue of abnormal proliferation, cytoskeleton organisation, and cell survival in CADASIL iPSC-derived iVSMCs by SNAP and sildenafil. IPSCs from CADASIL patient with *NOTCH3* C244Y mutation and its isogenic controls (C244Y_isoCtrl) were differentiated into iVSMCs via the neural crest lineage and treated with either the nitric oxide (NO) donor SNAP or the phosphodiesterase 5 (PDE5) inhibitor Sildenafil. (**A**) Proliferation was assessed by Ki67 immunofluorescence staining in the presence or absence of SNAP or sildenafil. Scale bar = 100 µm. (**B**) Cytoskeleton organisation of iVSMCs was visualised by F-actin staining treated with or without SNAP or Sildenafil. Scale bar = 5 µm. (**C**) Cell viability was assessed using live/dead staining with Cal-AM (green) indictive of live cells and BOBO-3 iodide (red) indicating dead cells. Scale bars = 100 µm**.** Nuclei were counterstained by DAPI.

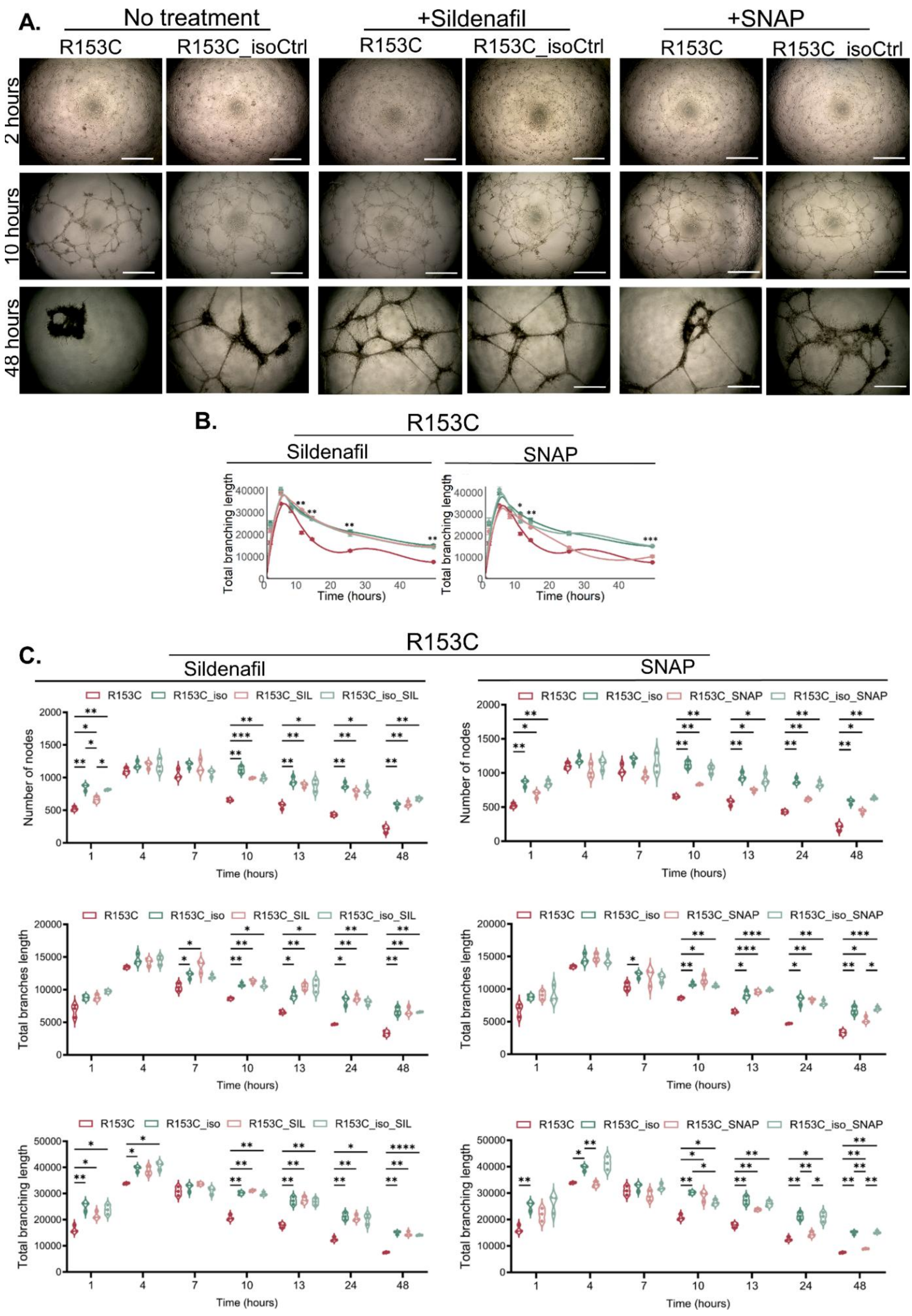
A.
No treatment
+Sildenafil
+SNAP
R153C
R153C_isoCtrl
2 hours
10 hours
48 hours
B.
R153C
Sildenafil
SNAP
Total branching length
Time (hours)
C.
R153C
Sildenafil
SNAP
R153C
R153C_iso
R153C_SIL
R153C_iso_SIL
R153C_SNAP
R153C_iso_SNAP
Number of nodes
Total branches length
Total branching length
Time (hours)

Fig. S12. SNAP and Sildenafil rescue impaired angiogenic support by *NOTCH3*-R153C iPSC derived iVSMCs. IPSCs from a CADASIL patient carrying the NOTCH3 R135C variant and the corresponding isogenic control line (R153C_isoCtrl) were differentiated into iVSMCs via neural crest lineage. The iVSMCs were co-cultured with HUVECs in a 1:2 ratio and subjected to angiogenesis in Matrigel for 48 hours to assess their ability to support network formation. (**A**) Representative light microscopy images of angiogenic structures formed under each condition. Scale bar = 1 mm. (**B**) Quantification of "total branching length" was shown as the fitted smooth trend lines (LOESS/liner) with 95% confidence interval. (**C**) Quantification of angiogenesis results at indicated time points using "Angiogenesis Analysis" plugin in ImageJ, based on number of nodes, total branches length, and total branching length. Data are mean ± SEM from 3 independent iPSC differentiations (n=3). Statistical significance was determined using two-way ANOVA followed by Tukey's post hoc test, $^{*}p \leq 0.05$, $^{**}p \leq 0.01$, $^{***}p \leq 0.001$, and $^{****}p \leq 0.0001$.

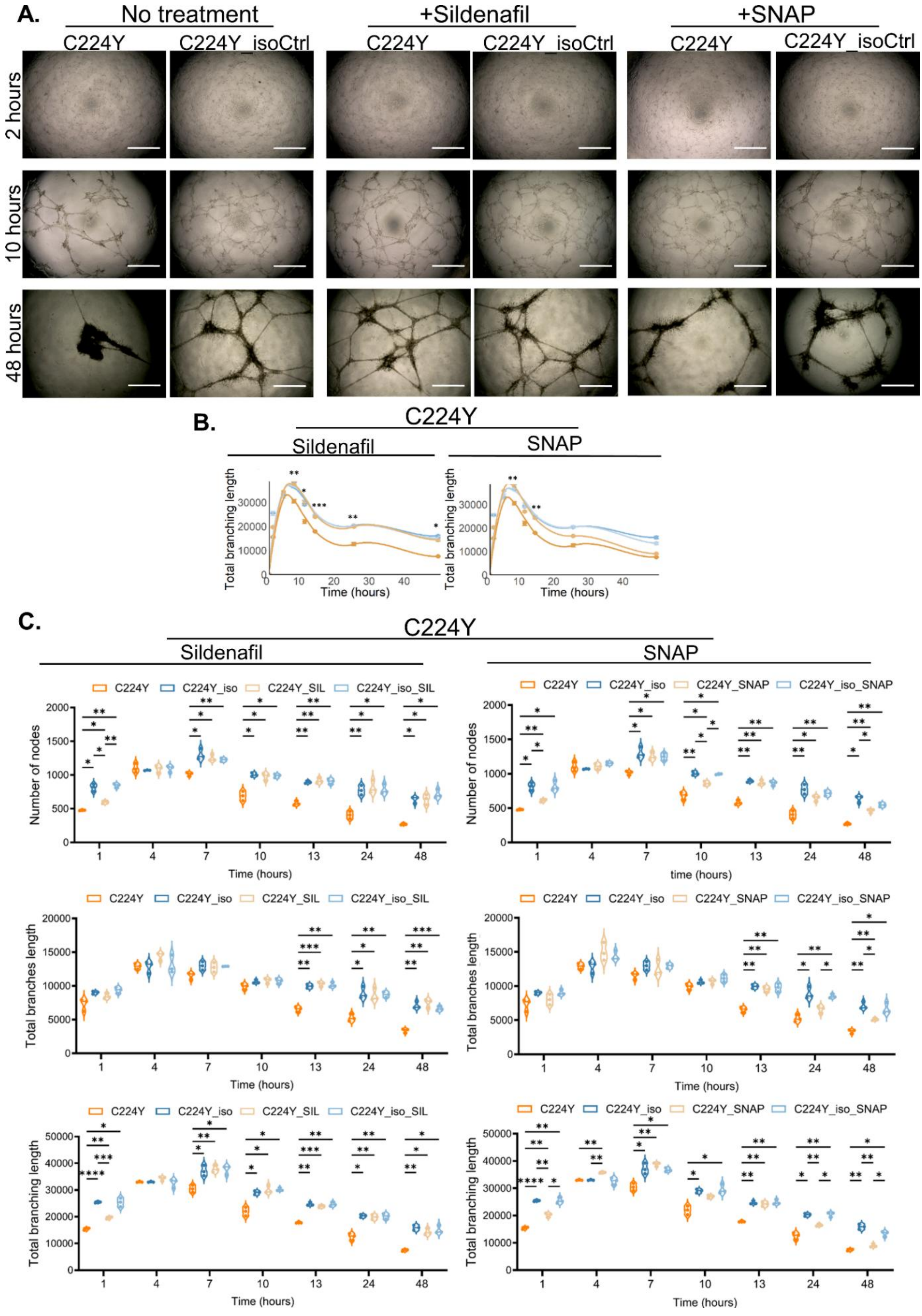
A.
No treatment
+Sildenafil
+SNAP
C224Y
C224Y_isoCtrl
2 hours
10 hours
48 hours
B.
C224Y
Sildenafil
SNAP
Total branching length
Time (hours)
C.
C224Y
Sildenafil
SNAP
C224Y
C224Y_iso
C224Y_SIL
C224Y_iso_SIL
C224Y_SNAP
C224Y_iso_SNAP
Number of nodes
Total branches length
Total branching length
Time (hours)
time (hours)

Fig. S13. SNAP and Sildenafil rescue impaired angiogenic support by *NOTCH3*-C224Y iPSC-derived iVSMCs. IPSCs from a CADASIL patient carrying the NOTCH3 C224Y variant and corresponding isogenic control line (C224Y_isoCtrl) were differentiated into iVSMCs via neural crest (NC) lineage. The iVSMCs were mixed with HUVECs in a 1:2 ratio and subjected to angiogenesis in Matrigel for 48 hours to determine the capability of iVSMCs in supporting the angiogenic network structure. (**A**) Images of the angiogenic network formation under light microscope in each condition. Scale bar = 1 mm. (**B)** Quantification of "total branching length" was shown as the fitted smooth trend lines (LOESS/liner) with 95% confidence interval. (**C**) Quantification of angiogenesis results at indicated time points using "Angiogenesis Analysis" plugin in imageJ, based on number of nodes, total branches length, and total branching length. Data are mean ± SEM from 3 independent iPSC differentiations (n=3). Statistical significance was determined using two-way ANOVA followed by Tukey's post hoc test, *$p \leq 0.05$, **$p \leq 0.01$, ***$p \leq 0.001$, and ****$p \leq 0.0001$.

Table S1. PCR primers used in the study.

Primer	Forward	Reverse	Efficiency
GAPDH	CATGTTCGTCATGGGTGTGAACCA	ATGGCATGGACTGTGGTCATGAGT	94.92
ACLP	ACCCACACTGGACTACAATGA	GTTGGGGATCACGTAACCATC	96.06
ACTA2	TGACAATGGCTCTGGGCTCTGTAA	TTCGTCACCCACGTAGCTGTCTTT	100.8
ACTG2	ATTGTGCGAGACATCAAGGAG	CCATGCCAATAAAGGAAGGCT	95.26
ACTN4	GCAGCATGGGCGACTACAT	TTGAGCCCGTCTCGGAAGT	94.25
CDH13	AGTGTTCCATATCAATCAGCCAG	CGAGACCTCATAGCGTAGCTT	88.90
CDH2	TGCGGTACAGTGTAACTGGG	GAAACCGGGCTATCTGCTCG	100.54
CNN1	GTCCACCCTCCTGGCTTT	AAACTTGTTGGTGCCCATCT	99.21
COL1A1	ATCAACCGGAGGAATTTCCGT	CACCAGGACGACCAGGTTTTC	108.49
COL1A2	GTTGCTGCTTGCAGTAACCTT	AGGGCCAAGTCCAACTCCTT	87.31
COL2A1	TGGACGCCATGAAGGTTTTCT	TGGGAGCCAGATTGTCATCTC	105.8
COL3A1	TTGAAGGAGGATGTTCCCATCT	ACAGACACATATTTGGCATGGTT	97.20
COL4A1	GGACTACCTGGAACAAAAGGG	GCCAAGTATCTCACCTGGATCA	106.67
COL4A2	TTATGCACTGCCTAAAGAGGAGC	CCCTTAACTCCGTAGAAACCAAG	99.25
FN	CGGTGGCTGTCAGTCAAAG	AAACCTCGGCTTCCTCCATAA	98.19
GUCY1A3	TCAGCCCTACTTGTTGTACTCC	CAGAATAGCGATGTGGGAATCAC	89.27
GUCY1B3	TGCTGGTGATCCGCAATTAC	CCAGGACACGCAAGATTGTATC	96.28
HAND1	CCATGCTCCACGAACCCTTC	CCTGGCGTCAGGACCATAG	99.28
HOX10A	CTCGCCCATAGACCTGTGG	GTTCTGCGCGAAAGAGCAC	92.74
HOX6C	ACAGACCTCAATCGCTCAGGA	AGGGGTAAATCTGGATACTGGC	88.08
ICAM	GTATGAACTGAGCAATGTGCAAG	GTTCCACCCGTTCTGGAGTC	100.08
ISL1	AGATTATATCAGGTTGTACGGGATC	ACACAGCGGAAACACTCGAT	87.79
ITGA1	GTGCTTATTGGTTCTCCGTTAGT	CACAAGCCAGAAATCCTCCAT	89.91
ITGA2	AGGTGGGGTTAATTCAGTATGCC	GATGTCTGGGATGTTGCTACAA	95.03
ITGA3	TCAACCTGGATACCCGATTCC	GCTCTGTCTGCCGATGGAG	100.54
ITGA4	CACAACACGCTGTTCGGCTA	CGATCCTGCATCTGTAAATCGC	92.86
ITGA5	AGACATTCGATCCCTCTACAACT	AATCGGCCAAACTCATCATGG	103.00
ITGA6	ATGCACGCGGATCGAGTTT	TTCCTGCTTCGTATTAACATGCT	98.68
ITGA7	CTGACTCCATGTTCGGGATCA	CACCTGTGAAGGTTTGGCG	100.42
ITGB1	CCTACTTCTGCACGATGTGATG	CCTTTGCTACGGTTGGTTACATT	90.77
ITGB3	CATGAAGGATGATCTGTGGAGC	AATCCGCAGGTTACTGGTGAG	86.63
MMP3	CGGTTCCGCCTGTCTCAAG	CGCCAAAAGTGCCTGTCTT	83.65
MMP9	TGTACCGCTATGGTTACACTCG	GGCAGGGACAGTTGCTTCT	86.92
MSN	GAGGATGTGTCCGAGGAATTG	GTCTCAGGCGGGCAGTAAA	90.26
MYH11	GACTTCCCTGCTCAATGCCT	GGACCTCTTCTCGTGGTTGG	101.65
MYH10	TGGTTTTGAGGCAGCTAGTATCA	AGTCCTGAATAGTAGCGATCCTT	99.28
MYL6	ACCAGACCGCAGAGTTCAAGGAG	CTCAAAGTCCAGCACCTTCACATT	91.99
MYL9	CCACATCCAATGTCTTCGCAATG	TGAAGTTGCCTTTCTTATCAATGGG	89.74
MYLK	GAGGTGCTTCAGAATGAGGACG	GCATCAGTGACACCTGGCAACT	109.21
MYOCD	CCACCTATGGACTCAGCCTAC	CTCAGTGGCGTTGAAGAAGAG	100.20
OCT4	AGACCATCTGCCGCTTTGAG	GCAAGGGCCGCAGCTT	91.63
PRKG1	CTTGGAGCTGTCGCAGATCC	TCTTTGATGATGCAACTGTCCTT	97.21
RBP1	CGCACGCTGAGCACTTTTAG	GCACTTGCGGTCATCTATGC	105.26
SEMA3a	GTGCCAAGGCTGAAATTATCCT	CCCACTTGCATTCATCTCTTCT	90.04
SMTN	CGGCTGCGCGTGTCTAATCC	CTGTGACCTCCAGCAGCTTCCG	98.84
SOX1	CCTCCGTCCATCCTCTG	AAAGCATCAAACAACCTCAAG	91.29
SPEG	AACCGCCGTTCTTCTGACAC	TGGTCCATAAGTGAGACCTTGAA	97.26
SPP1	CTCCATTGACTCGAACGACTC	CAGGTCTGCGAAACTTCTTAGAT	93.63
TAGLN	CGCGAAGTGCAGTCCAAAAT	CAGCTTGCTCAGAATCACGC	85.01
TBX6	CATCCACGAGAATTGTACCCG	AGCAATCCAGTTTAGGGGTGT	100.11
TIMP1	CCACCATGAGACCTCAACCC	GCCACTACAGCCGTATTCTCC	92.65
TIMP3	TGGCGCAGTGAGAACTTCG	CCCCGAGTAGAGGTCATCCAG	97.32
TFAP2A	AGGTCAATCTCCCTACACGAG	GGAGTAAGGATCTTGCGACTGG	85.75
VASP	ATGGCAACAAGCGATGGCT	CGATGGCACAGTTGATGACCA	103.21

Appendix 1. Whole blot images from western blots.

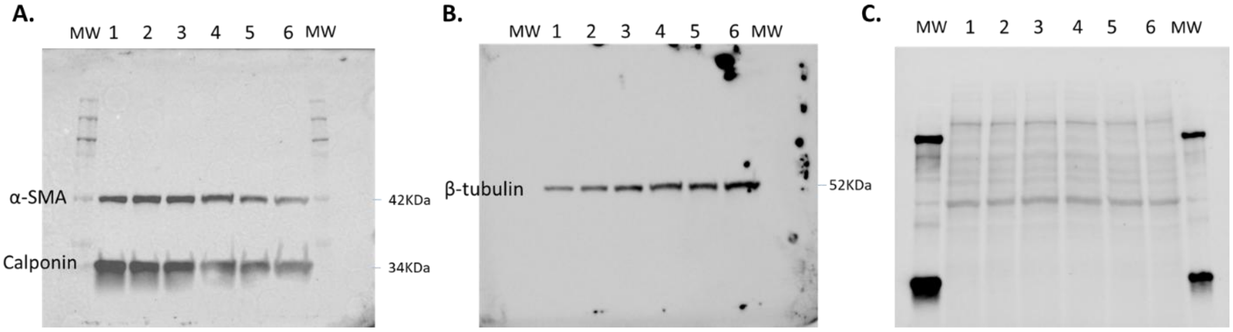

Fig. App1. An example of whole blot images of western blot. IPSC-derived VSMCs were subjected to western blot. **A.** The blot was co-probed simultaneously by antibodies against α-SMA (Abcam, ab7817) and calponin (Abcam, rabbit). **B.** The antibodies were stripped off, and the blot was re-probed by β-tubulin. However, the β-tubulin staining was uneven from left to right as compared to the total protein loading (**C**) of the same blot. Therefore, the whole blot was not used in the subsequent experiments. Samples on lanes 1-6 were: isoCtrl1, isoCtrl2, isoCtrl3, C224Y1, C224Y2, and C224Y3, in order. MW, molecular weight markers. This figure relates to Figure 3D.

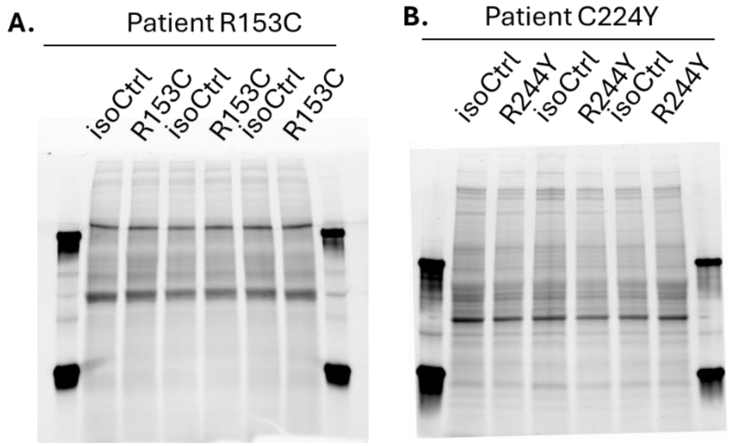

Fig. App2. An example of whole blot images of western blot. IPSC-derived VSMCs were subjected to western blot. Images of total protein loading are shown in **A** (R153C) and **B** (C224Y). This figure corelates to Figure S10.
